# Using expert-modeling videos in telesimulations to teach pediatric and neonatal nursing *

**DOI:** 10.1590/1518-8345.7044.4242

**Published:** 2024-11-22

**Authors:** Mariane Caetano Sulino Gonçalves, Aline Natalia Domingues, Luciana Mara Monti Fonseca, Regina Aparecida Garcia de Lima, Larissa Martiano de Lima, Aline Cristiane Cavicchioli Okido, Juliana Coelho Pina, Adriana Moraes Leite, Lucila Castanheira Nascimento, Maria Cândida de Carvalho Furtado

**Affiliations:** ^1^ Universidade de São Paulo, Escola de Enfermagem de Ribeirão Preto, PAHO/WHO Collaborating Centre for Nursing Research Development, Ribeirão Preto, SP, Brazil.; ^2^ Scholarship holder at the Coordenação de Aperfeiçoamento de Pessoal de Nível Superior (CAPES), Brazil.; ^3^ Universidade de São Paulo, Escola de Enfermagem de Ribeirão Preto, Departamento de Enfermagem Materno-Infantil e Saúde Pública, PAHO/WHO Collaborating Centre for Nursing Research Development, Ribeirão Preto, SP, Brazil.; ^4^ Scholarship holder at the Conselho Nacional de Desenvolvimento Científico e Tecnológico (CNPq), Brazil.; ^5^ Universidade Federal de São Carlos, Departamento de Enfermagem, São Carlos, SP, Brazil.; ^6^ Universidade Federal de Santa Catarina, Departamento de Enfermagem, Florianópolis, SC, Brazil.

**Keywords:** Pediatric Nursing, Neonatal Nursing, Instructionnal Film and Video, Education, Nursing, Simulation Training, Nursing Education Research

## Abstract

to assess the perception of self-efficacy, self-confidence, and satisfaction of undergraduate nursing students during the use of expert-modeling videos as a learning strategy in the period of social distancing.

this was a descriptive study with undergraduates studying disciplines of caring for hospitalized newborns and children at a higher education nursing institution in the interior of the state of São Paulo, Brazil. Activities were organized in three moments, in real time: prebriefing, expert-modeling videos and debriefing. The expert-modeling videos replaced the clinical simulation scenario during the social distancing required by the COVID-19 pandemic, for the development of telesimulation. Specific validated scales were used to analyze students’ self-confidence, self-efficacy and satisfaction. Content analysis assessed the advantages and disadvantages of using expert-modeling videos.

thirty-nine undergraduates took part in the study. High levels of self-confidence, self-efficacy and satisfaction were found among the participants. The advantages of expert-modeling videos were organized into the following categories: Convenience, Security and continuity of studies and Efficient teaching method, and the disadvantages into Problems with technology, Distractions, Lack of personal contact and Mental fatigue.

telesimulation activities were consolidated as strategies that enabled learning in times of emergency online teaching due to the realness of telesimulation, the development of skills in caring for children and newborns and the link between theory and practice.

## Highlights

**(1)** Innovative learning strategy using expert-modeling videos.

**(2)** Self-efficacy and self-confidence of undergraduates with telesimulation in child health.

**(3)** Satisfaction with the use of telesimulation for teaching pediatric and neonatal nursing.

**(4)** Advantages of using expert-modeling videos in times of social distancing.

**(5)** The strategy showed positive results in the teaching-learning process.

## Introduction

 With the closing of higher education institutions (HEIs) during the COVID-19 pandemic in early 2020 ^(1)^, due to the necessity of observing social distancing, students and professors of undergraduate nursing courses faced challenges in the teaching-learning process. At the time, it was necessary to look for new forms of education to maintain the quality required for the training process focused on care centered on the individual and the community, with a view to training an autonomous and proactive professional, with critical-reflective thinking, clinical reasoning, decision-making skills and discernment to act in the job market ^(2)^.

As a strategy for continuing the teaching-learning process, since the beginning of the pandemic most of the activities have been carried out remotely, on online platforms such as Google Meet, Zoom, Skype, and Moodle, among others.

 In this context, simulations have stood out as an active methodology that enables students to develop meaningful learning, made possible by experiencing realistic situations in a safe and risk-free environment ^(3)^. Students can participate in the simulated environment as role-players or active observers; in both cases, they have the opportunity to learn technical ^(4)^ and non-technical skills, either by carrying out actions or by paying attention to the information available in the scenario and the activities carried out by the participants ^(5)^. Studies show that the use of simulation has a positive and significant impact on nursing education when compared to traditional education ^(4), 6 - 9)^.

 Studies comparing clinical judgment between student participants, role-players and observers have found varying levels of analysis and understanding of the simulated clinical case, but in general, the results show that participation as an observer also provides significant learning results ^(5, 10 - 11)^.

 Telesimulation is defined as a teaching-learning process that uses telecommunication and simulation resources to provide education, training and assessments for students in an external location, when there are some geographical limitations, for example ^(12)^. This teaching model includes expert-modeling videos, which are used to prepare students by putting them in the role of observers. The expert-modeling videos can be staged by the professors themselves and nurses who are experts in the topic discussed on stage, in a realistic clinical scenario, in which the actors perform tasks or clinical care, according to the proposed topic and the learning objectives ^(13)^. 

 Studies show that the use of expert-modeling videos in the classroom as a teaching-learning method, placing the student as an observer, improves their clinical judgment skills ^(5, 14 - 15)^.

Expert-modeling videos in telesimulation were also used to teach care of hospitalized newborns and children during the shutdown period of nursing teaching institutions, in which face-to-face clinical simulations also had to be suspended.

Thus, the aim of this study was to assess the perception of self-efficacy, self-confidence and satisfaction of undergraduate nursing students when using expert-modeling videos as a learning strategy for caring for hospitalized newborns and children during social distancing.

## Method

### Study design

This was a descriptive study with the aim of gauging the perception of undergraduate nursing students regarding aspects of self-confidence, self-efficacy and satisfaction with the use of expert-modeling videos as a teaching-learning strategy in telesimulation.

 This type of study aims to observe, describe and document aspects of a situation ^(16)^, in this case, the use of expert-modeling videos as a teaching-learning strategy. 

This study was reported according to recommendations from Strengthening the Reporting of Observational Studies in Epidemiology (STROBE).

### Study location

The study was carried out at a nursing Higher Education Institution (HEI) of a public university in the interior of the state of São Paulo, Brazil. The HEI offers bachelor’s and bachelor’s and licentiate degrees in nursing, and around 80 and 50 students enter the respective courses each year, totaling 130 available places.

### Scenario description

**Figure 1 -:**
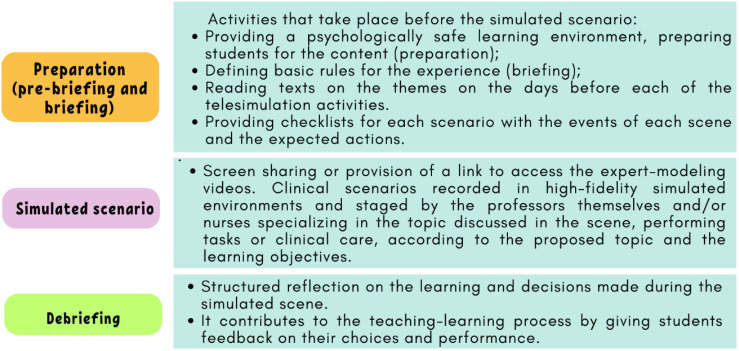
Stages for carrying out telesimulation as a teaching-learning strategy ^(17)^

 According to the International Nursing Association for Clinical Simulation and Learning (INACSL) ^(17)^ best practices in simulation, the simulated activities followed the stages of preparation (prebriefing and briefing), scenario in action, in this case the expert-modeling videos and debriefing (Figure 1). With the participation of students and professors, the prebriefing was held in real time, a screen was shared for the transmission of the expert-modeling videos and then the structured debriefing took place synchronously via the Google Meet platform. 

The expert-modeling videos were previously recorded at the Simulation Center and featured four different validated, high-fidelity simulated scenarios set up for practice with all the realistic environmental elements, materials, and equipment, medium- and high-fidelity simulators and trained role-players. These four simulated scenarios were developed and validated and have been used in courses for some years, prior to the pandemic; they deal with clinical conditions and complications, such as aspiration of diet/swallowing, hyperthermia, dehydration, and respiratory failure, as these are clinical conditions/complications commonly found in the context of pediatric and neonatal units.

### Population

Nursing undergraduates enrolled in the subjects of care for hospitalized newborns and children in the academic year 2021 participated in the study. The subjects are offered every semester, in the first semester for the bachelor’s and licentiate’s degree courses in nursing, with 50 places, and in the second semester for the bachelor’s degree course in nursing, with 80 places. During the 2021 academic year, the courses were offered remotely and synchronously via the Google Meet platform. All the students who took the courses in that academic year were invited to take part in the research. Those absent from class on the days the researcher collected the data were excluded from the study.

### Data collection

Data collection took place from April to June 2022, by a postgraduate student who accompanied all the moments in which the telesimulated activities with expert-modeling videos were carried out. Each of the four scenarios was carried out on different dates and organized into three moments: pre-briefing, expert-modeling videos, and debriefing, during the courses.

 The choice to start collecting data only after the subjects had finished was motivated by the intention of preventing a hierarchical or authoritative relationship from limiting the autonomy of the research participants ^(18)^. 

For data collection, the students were approached at the beginning of the activities of other subjects, with the authorization of the responsible professors. When inviting them, the postgraduate student responsible for the data collection informed them of the objectives of the research, the guarantee of voluntary participation, the risks, and benefits of taking part in the study, stressing that there would be no benefits or losses in the grades of the students who took part in the research.

 For those who were interested in taking part, an instrument organized into two parts was given to them. The first contained two copies of the informed consent form, a characterization instrument with information such as gender, age, course (bachelor’s degree or bachelor’s degree and licentiate), whether the participant had had previous contact with simulation, how they participated in the simulation, the types of technological device they used and an open field for listing advantages and disadvantages of using expert-modeling videos. The second part included the Student Satisfaction and Self-Confidence in Learning Scale, developed by the National League for Nursing (NLN), a league dedicated to excellence in nursing education and validated in Brazil ^(19)^, and the Self-Efficacy Scale ^(20)^, developed from Bandura’s social learning theory by Brazilian researchers. Both are self-administered and comprise 13 items each, assessed using a Likert-type scale with five response options (ranging from 1 = strongly disagree to 5 = strongly agree). The higher the total score in the sum of the items, the higher the levels of satisfaction, self-confidence and self-efficacy perceived by the student. A total of 39 undergraduates took part, 20 (51.3%) from the bachelor’s degree course and 19 (48.7%) from the bachelor’s and licentiate degree courses.

### Data analysis

 The characterization data and scale scores were double-entered into a database prepared using a Microsoft Excel ^®^ spreadsheet by two researchers, independently. SPSS Statistics version 22 was used for statistical analysis, with the help of a statistician from the higher education institution. Descriptive statistics included calculating the frequency, mean, median, standard deviation, minimum and maximum of the variables investigated. 

 Content analysis was used to analyze the answers to the open questions regarding advantages and disadvantages ^(21)^. This type of analysis allows the researcher to quantify and qualify the data. To this end, the stages of material exploration, categorization and interpretation were followed. This process was carried out by four researchers of the group, two of whom were individually responsible for categorizing the advantages and disadvantages and then meeting to review them, analyze the discrepancies and reach a consensus. The categories were then validated by two other researchers. Thus, the advantages were organized into three categories: Convenience; Security and continuity of studies; and Efficient teaching method. The disadvantages were grouped into four categories: Problems with technology; Distractions; Lack of personal contact; and Mental fatigue.

### Ethical considerations

 The study was conducted in accordance with the Declaration of Helsinki and approved by the higher education institution’s Research Ethics Committee (Certificate of Presentation for Ethical Consideration 42613021.2.0000.5393). All participants involved in the study were informed about the objectives of the research and other aspects set out in CNS Resolutions 466 of 2012 ^(22)^ and 510 of 2016 ^(18)^. All information was protected to guarantee anonymity and used exclusively for research purposes. 

## Results

A total of 39 students took part in the study, 89.7% (n = 35) of whom were female, 7.7% (n = 3) male and 2.6% (n = 1) who preferred not to declare their gender. Regarding age, the average was 21 years, with 25.6% (n = 10) being 36 years old at the most and 20 years old at the least. All the students had already had previous contact with the use of active methodologies and information technologies, as well as taking part in simulation activities during their undergraduate studies before the pandemic hit.

Table 1 shows data on participant satisfaction and self-confidence in learning. On the axis of satisfaction with current learning, the items with the highest frequency were 1 ( *The teaching methods used in this simulation were useful and effective*), with 76.9% (n = 30) frequency of responses, followed by item 5 (*The way my professor taught through the simulation was appropriate for the way I learn*), with 61.5% (n = 24). 

 The items that stood out the most on the self-confidence axis were 7 (*I am confident that this simulation included the content necessary to master the pediatric nursing curriculum*) and 9 (*My professor employed useful resources to teach the simulation*), both with a 56.4% frequency of responses (n = 22). 

**Table 1 -:** Student satisfaction and self-confidence about learning. Ribeirão Preto, SP, Brasil, 2022

	**Satisfaction with current learning**	**1**	**2**	**3**	**4**	**5**
1	The teaching methods used in this simulation were useful and effective.	2.6% (n=1)	7.7% (n=3)	7.7% (n=3)	76.9% (n=30)	5.1% (n=2)
2	The simulation provided me with a variety of teaching materials and activities to promote my learning of the pediatric nursing curriculum.	-	12.8% (n=5)	15.4% (n=6)	51.3% (n=20)	20.5% (n=8)
3	I liked the way my professor taught through the simulation.	-	5.1% (n=2)	12.8% (n=5)	59.0% (n=23)	23.1% (n=9)
4	The materials used in this simulation were motivating and helped me learn.	-	7.7% (n=3)	23.1% (n=9)	51.3% (n=20)	17.9% (n=7)
5	The way my professor taught through the simulation was appropriate for the way I learn.	-	7.7% (n=3)	15.4% (n=6)	61.5% (n=24)	15.4% (n=6)
	**Self-confidence in learning**					
6	I am confident that I have mastered the content of the simulation activity that my professor presented to me.	2.6% (n=1)	17.9% (n-7)	33.3% (n=13)	38.5% (n=15)	7.7% (n=3)
7	I am confident that this simulation included the content necessary to master the pediatric nursing curriculum.	-	12.8% (n=5)	17.9% (n=7)	56.4% (n=22)	12.8% (n=5)
8	I am confident that I am developing skills and gaining the necessary knowledge from this simulation to perform the necessary procedures in a clinical setting.	-	17.9% (n=7)	15.4% (n=6)	48.7% (n=19)	17.9% (n=7)
9	My professor employed useful resources to teach the simulation.	-	2.6% (n=1)	12.8% (n=5)	56.4% (n=22)	28.2% (n=11)
10	It is my responsibility as a student to learn what I need to know through the simulation activity.	-	12.8% (n=5)	28.2% (n=11)	28.2% (n=11)	30.8% (n=12)
11	I know how to get help when I do not understand the concepts covered in the simulation.	-	5.1% (n=2)	2.6% (n=1)	46.2% (n=18)	46.2% (n=18)
12	I know how to use simulation activities to learn skills.	2.6% (n=1)	5.1% (n=2)	12.8% (n=5)	51.3% (n=20)	28.2% (n=11)
13	It is the professor’s responsibility to tell me what I need to learn about the topic developed in the simulation during the lesson.	-	17.9% (n=7)	38.5% (n=15)	30.8% (n=12)	12.8% (n=5)

When analyzing the total score, the mean was 43.8 and the median was 49, with a maximum score of 62, which shows a high level of satisfaction and self-confidence.

Table 2 shows the results of the Self-Efficacy Scale. The items that stood out most in this regard were 2 ( *I trust my abilities*), with a response frequency of 64.1% (n = 25), 3 (*When I decide to do something, I immediately take action* ), with 51.3% (n = 20), 4 (*I cope well with unexpected problems*), also with 51.3% (n = 20), 6 (*I see difficulties as a challenge*), also with 51.3% (n = 20), and 13 (*I recover quickly after a failure*), with a frequency of 51.3% (n = 20). 

In the total score for self-efficacy, there was an average of 41.5 points and a median of 42, with a maximum score of 52, showing a high level, in the students’ perception.

**Figure 2:**
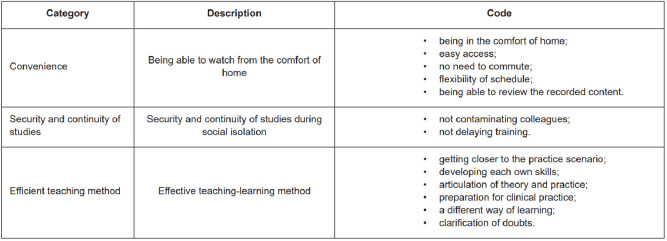
- Advantages of using expert-modeling videos in teaching and learning pediatric and neonatal nursing. Ribeirão Preto, SP, Brasil, 2022

**Figure 3:**
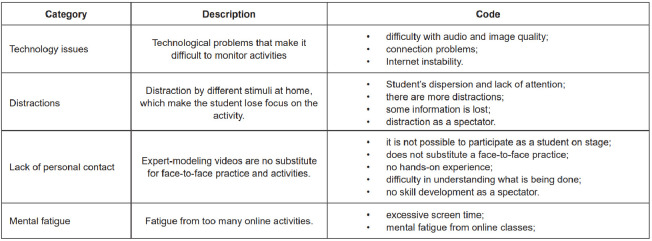
- Disadvantages of using expert-modeling videos in pediatric and neonatal nursing education. Ribeirão Preto, SP, Brasil, 2022

 Students were asked to indicate the advantages (Figure 2) and disadvantages (Figure 3) of using the expert-modeling videos strategy for learning pediatric and neonatal nursing. The answers were categorized by similarity and described below. 

**Table 2:** - Self-efficacy Scale (n = 39). Ribeirão Preto, SP, Brasil, 2022

	**Self-efficacy Scale (SES)**	**1**	**2**	**3**	**4**	**5**
1	I am capable of successfully carrying out my life plans.	-	-	25.6% (n=10)	46.2% (n=18)	28.8% (n=11)
2	I trust my abilities.	-	7.7% (n=3)	17.9% (n=7)	64.1% (n=25)	10.3 (n=4)
3	When I decide to do something, I immediately take action.	-	5.1% (n=2)	23.1% (n=9)	51.3% (n=20)	20.5% (n=8)
4	I cope well with unexpected problems.	-	15.4% (n=6)	30.8% (n=12)	51.3% (n=20)	2.6% (n=1)
5	I feel able to cope well with most of the problems that arise in my life.	-	10.3% (n=4)	35.9% (n=14)	38.5% (n=15)	15.4% (n=6)
6	I see difficulties as a challenge.	2.6% (n=1)	17.9% (n=7)	12.8% (n=5)	51.3% (n=20)	15.4% (n=6)
7	I give up easily on what I set out to do.	48.7% (n=19)	35.9% (n=14)	5.1% (n=2)	10.3% (n=4)	-
8	If something seems too complicated, I do not even try.	59.0% (n=23)	23.1% (n=9)	10.3% (n=4)	7.7% (n=3)	-
9	I feel insecure in the face of failure.	10.3% (n=4)	15.4% (n=6)	25.6% (n=10)	33.3% (n=13)	15.4% (n=6)
10	I get overwhelmed by failures.	20.5% (n=8)	23.1% (n=9)	25.6% (n=10)	25.6% (n=10)	5.1% (n=2)
11	I feel incapable of carrying out a new activity without instructions.	20.5% (n=8)	25.6% (n=10	28.7% (n=11)	20.5% (n=8)	5.1% (n=2)
12	I can say that I have had more successes than failures in my life.	-	5.1% (n=2)	30.8% (n=12)	48.7% (n=19)	15.4% (n=6)
13	I recover quickly after a failure.	-	10.3% (n=4)	35.9% (n=14)	51.3% (n=20)	2.6% (n=1)

### Advantages of expert-modeling videos in the teaching-learning process in pediatric and neonatal nursing

When asked about the advantages of using expert-modeling videos as a teaching-learning strategy, the students reported aspects such as the comfort of being able to learn at home and safe from COVID-19 contamination, the possibility of getting closer to practice scenarios even remotely, as well as the effectiveness of the strategy for learning. Their statements were categorized as follows:

 The students identified the possibility of attending classes from the comfort of their homes as an advantage. The Convenience category is present in statements such as: “*being in the comfort of home*”, “*saves time, because you don’t have to travel to college*”, “ *participating from another city*”, “*flexibility of schedule*”, “*possibility of reviewing the recorded content*” and “*possibility of watching it again* ”. 

 The safety of being protected from COVID-19 infection and the possibility of maintaining the teaching-learning process were also identified as advantages: “ *not missing out on activities even during social distancing* ”, “*allowing the course to run smoothly during the epidemic*” and “*not contaminating colleagues*”. 

 The methodology adopted brought students closer to the practice scenarios, as seen in some of the following statements: “*getting closer to reality*”, “*simulating what practice would be like*”, “*getting closer to the practice scenario* ” and “*making it easier to understand the theoretical content and answer questions*”. 

 Moreover, the statements suggest that expert-modeling videos can be an efficient teaching-learning strategy because they provide different experiences, such as: “*preparation for clinical practice*”, “*development of each own skills*”, “*using different strategies with students*” and “*different way of learning*”. 

 Most of the positive statements were related to the method used to continue the teaching-learning process, a sign that expert-modeling videos can be a powerful strategy to be used not only in periods when classes cannot be held in person, as in the case of the pandemic, but also as an additional strategy to the class models already adopted. In this sense, some possibilities emerged for the use of this tool: “*clarification of doubts*”, “*for clarity of procedures*” and “*provides a first contact similar to reality*”. 

### Disadvantages of using expert-modeling videos in teaching and learning pediatric and neonatal nursing

As disadvantages, the students pointed to aspects related to technological problems, easy distraction by different stimuli at home, tiredness due to too many online classes and the impossibility of practicing the content discussed in class. Based on this, the following categories are presented below.

 Technical problems were cited as disadvantages to using the proposed method, in view of the statements about the overload of the Internet network, such as “ *Internet instability*”, “*difficulty accessing the Internet*”, “*if the connection goes down it gets in the way*”, and regarding the quality of the images and audio of the videos used: “*depends on a good Internet for a good image*”, “ *difficulty with audio and image quality*”, “*bad microphone made it difficult to understand*” and “*poor video framing*”. 

 Despite the fact that being in the comfort of home was mentioned as an advantage, the distracting factors that this environment provides were listed as a disadvantage, as they take the focus away from the teaching activity: “ *easy dispersion”,*”*there are more distractions*”, “*student’s* *dispersion and lack of attention*”, ”*distraction as a spectator*”, *“some information is lost”* and *“not as much attention*”. However, these disadvantages can be offset by the possibility of reviewing the recorded content, an aspect that was also mentioned as an advantage. 

 In addition, the students pointed out the lack of interaction with the practical content as a disadvantage: “*it doesn’t replace the practical/presential”,*”*it doesn’t develop skills as a spectator*”, “*you don’t have your hands on*” and *“it’s not practical for the student*”. This type of disadvantage is relevant, as it can interfere with students’ satisfaction and self-confidence in the learning process, which would not occur if it were a strategy to add to the different teaching strategies that already exist, including practice in real social scenarios. In this sense, the moment of social distancing gave the students a feeling of losing the clinical component of real practice. 

 Mental fatigue was also cited as a disadvantageous factor when using telesimulation with videos as a teaching-learning strategy. This was evidenced in the following statements: *“mental fatigue in the face of online classes”* and *“fatigue due to too many online activities on the computer”.* These considerations stem from the context in which all the students were living during the data collection period, i.e., social distancing due to the COVID-19 pandemic, when teaching became totally online. In summary, it can be highlighted that nursing students considered that the expert-modeling videos method has advantages in situations where it is not possible to carry out activities in person for safety reasons, such as in the context of a pandemic. Moreover, it can be used as a strategy to review and consult the content discussed in the subjects, but it does not replace classroom teaching and clinical practice in social settings. 

## Discussion

 Telesimulation in the teaching-learning process in neonatal and pediatric nursing has proven to be attractive and effective, especially during emergency online teaching. In recent years, health education has undergone significant transformations, with emphasis on education mediated by clinical simulation ^(23)^. The use of telesimulation to instruct complex theoretical-practical scenarios is a relatively new approach, as is involving all students in their residencies, providing an authentic and immersive experience, with real cases and opportunities to practice in a clinical environment ^(24)^. Telesimulation debriefing has also been shown to be effective in increasing students’ perception of simulation effectiveness and should be integrated into nursing simulation experiences whenever possible ^(25)^. 

 The use of telesimulation to teach a complex case-based theoretical-practical scenario is relatively new, as is working with all students located in their homes, as it involves providing an authentic and immersive mode, with a real case and the opportunity to practice actions in a clinical scenario ^(24)^. Telecommunication debriefing itself helps to increase the students’ perception of the effectiveness of the simulation and should be incorporated into nursing simulation experiences as much as possible ^(25)^. 

 In this sense, in the present study, the telesimulation scenario brought aspects of a triangulation of contexts, seeking to interact with the students in their homes and the professors in conducting the telesimulation. The benefits of telesimulation extend beyond simulation centers and are valid where there are distance limitations that prevent effective and efficient instruction in a given practice, as well as in complex scenarios that require several repetitions in the laboratory ^(26)^. 

 In this study, the telesimulation scenario was designed to interact with the students at home, while the professors conducted the telesimulation, resulting in a triangulation of contexts. The benefits of telesimulation go beyond the limits of simulation centers and are especially relevant in situations where distance makes effective instruction difficult and in complex scenarios that require repeated practice in the laboratory ^(26)^. 

 In this study, the telesimulation scenario was designed to interact with the students at home, while the professors conducted the telesimulation, resulting in a triangulation of contexts. The benefits of telesimulation go beyond the limits of simulation centers and are especially relevant in situations where distance makes effective instruction difficult and in complex scenarios that require repeated practice in the laboratory ^(26)^. The COVID-19 pandemic has significantly altered teaching and learning, and telesimulation has emerged as a crucial alternative for maintaining continuity in undergraduate education, creating robust online educational experiences that maximize learning opportunities ^(27)^. 

 The context of the COVID-19 pandemic has led to changes in everyday teaching and learning, and telesimulation has emerged as a crucial alternative to maintain the continuity of undergraduate education and to create robust online educational experiences that maximize learning opportunities ^(27)^, similar to what was found in this study. 

 Another aspect is that most of the students were satisfied with telesimulation in the expert-modeling video modality, a result also identified in other studies, with a perception of greater engagement and encouragement to think critically ^(24 - 25)^. They also mentioned the flexibility and ease of discussion and of learning both by observing and getting actively involved. 

 In this way, telesimulation can be credited with extending the benefits of teaching practices beyond laboratories and simulation centers, with several advantages. Some of these include accessibility, economy, increased knowledge, maintenance of activities, satisfaction with learning, use of simulation in remote locations, improvement of skills, improved interaction, interdisciplinary interaction, increased confidence, comfort, and lower risk of contamination ^(6)^. 

 Studies also highlight limitations in carrying out online activities, due to audio failures ^(24)^ and distractions and limited Internet connection, similar to the challenges addressed in this study. The creation of scenarios that resemble reality, in order to capture concrete elements, played an important role in this research and generated new insights as a result of this debriefing process. This moment of reflection revealed valuable ideas for developing perspectives on professional practices and healthcare. Debriefing is widely recognized as effective in providing enriching experiences and learning opportunities for students, and is used in simulation for nursing education to improve clinical skills and learning outcomes ^(28)^. 

 The experiences that nursing students have with the use of technologies and active strategies during their undergraduate studies are seen as a possibility for diversified ways of learning and, at the same time, preserving a safe and realistic environment. In addition, they allow us to get closer to telehealth, which is growing in clinical practice and linked to an increase in healthcare ^(29 - 30)^. Another important factor is that role demonstrations through modeling videos with experts can also reduce student anxiety and improve preparation for simulated learning experiences ^(29)^. 

 The creation of realistic scenarios played a crucial role in the telesimulation, allowing for in-depth reflection that generated valuable insights for developing perspectives on professional practices and healthcare. Debriefing is recognized as an effective tool for providing enriching learning experiences and is widely used in simulation for nursing education to improve clinical skills and learning outcomes ^(28)^. 

 Nursing students’ experiences with technologies and active strategies during their undergraduate studies not only diversify the ways in which they learn, but also prepare them for an increasingly connected professional environment. In addition, these experiences bring them closer to telehealth, a growing trend in clinical practice, associated with advances in healthcare ^(29)^. 

Some limitations were identified in this study, for example, all the students enrolled in the subjects of care for hospitalized newborns and children were invited to take part in the research, out of a total of 130, of which only 30% agreed to take part. This can be attributed to the fact that, at the time, many students were already taking part in numerous online activities and some of them were overloaded with these undergraduate demands.

 It is also possible that the high levels of self-efficacy, self-confidence and satisfaction measured in this study are related to the participants’ exposure to simulation activities prior to the course and the pandemic. Motivation is crucial for effective learning, and the application of active methodologies effectively strengthens the skills acquired by students during the learning process ^(30)^. 

 It is worth noting that the results obtained in this study highlight the relevance of discussing and adopting information and communication technologies (ICT) in the teaching of nursing courses, since they are common elements in the daily lives of undergraduate students today, as well as being able to support them in their autonomy in the search for knowledge and the apprehension of content ^(31)^. 

## Conclusion

The study showed that telesimulation activities were consolidated as strategies that enabled learning in times of emergency online teaching, due to the realness of telesimulation, the development of skills in caring for children and neonates and the link between theory and practice. Prebriefing and debriefing in real time, both through telecommunications (videoconferencing), proved to be another important strategy in the execution of telesimulations.

In the students’ perception, the use of the expert-modeling videos strategy in telesimulation activities allowed for learning in times of emergency online teaching, comfort in being at home, the realness of telesimulation with practice, the development of skills in caring for children and newborns and the articulation between theory and practice. On the other hand, the disadvantages were problems connecting to the Internet network, distraction due to being at home, tiredness due to too many classes and online activities and the impossibility of applying the theoretical content in face-to-face practical activities in real clinical scenarios.

Despite the disadvantages, the benefits of the expert-modeling video strategy in telesimulation activities stand out in terms of the students’ teaching-learning process, as well as increasing their level of self-confidence, self-efficacy, and satisfaction. The results of this study allow us to identify the advantages of using expert-modeling videos in telesimulation activities effectively and in a safe virtual environment, accompanied by facilitator professors throughout the activity.
