# Supplementary material for: Using expert-modeling videos in telesimulations to teach pediatric and neonatal nursing
Source: Rev Lat Am Enfermagem. 2024 Nov 22;32:e4242. doi: 10.1590/1518-8345.7044.4242 (PMC11654050; doi:10.1590/1518-8345.7044.4242)
Supplement: Translation

# Translation: Uso de *expert-modeling videos* en telesimulaciones para enseñar enfermería pediátrica y neonatal *

**DOI:** 10.1590/1518-8345.7044.4241

**Published:** 2024-11-15

**Authors:** Mariane Caetano Sulino Gonçalves, Aline Natalia Domingues, Luciana Mara Monti Fonseca, Regina Aparecida Garcia de Lima, Larissa Martiano de Lima, Aline Cristiane Cavicchioli Okido, Juliana Coelho Pina, Adriana Moraes Leite, Lucila Castanheira Nascimento, Maria Cândida de Carvalho Furtado

**Affiliations:** ^1^ Universidade de São Paulo, Escola de Enfermagem de Ribeirão Preto, Centro Colaborador de la OPS/OMS para el Desarrollo de la Investigación en Enfermería, Ribeirão Preto, SP, Brasil.; ^2^ Becaria de la Coordenação de Aperfeiçoamento de Pessoal de Nível Superior (CAPES), Brasil.; ^3^ Universidade de São Paulo, Escola de Enfermagem de Ribeirão Preto, Departamento de Enfermagem Materno-Infantil e Saúde Pública, Centro Colaborador de la OPS/OMS para el Desarrollo de la Investigación en Enfermería, Ribeirão Preto, SP, Brasil.; ^4^ Becaria del Conselho Nacional de Desenvolvimento Científico e Tecnológico (CNPq), Brasil.; ^5^ Universidade Federal de São Carlos, Departamento de Enfermagem, São Carlos, SP, Brasil.; ^6^ Universidade Federal de Santa Catarina, Departamento de Enfermagem, Florianópolis, SC, Brasil.

**Keywords:** Enfermería Pediátrica, Enfermería Neonatal, Película y Vídeo Educativo, Educacíon em Enfermeira, Entrenamiento Simulado, Investigación em Educacíon de Enfermería

## Abstract

medición de la percepción de autoeficacia, autoconfianza y satisfacción de estudiantes universitarios de enfermería durante el uso de *expert-modeling videos* como estrategia de aprendizaje en el periodo de distanciamiento social.

se trató de un estudio descriptivo con estudiantes que cursaban cuidados a recién nacidos y niños hospitalizados en una institución de enseñanza superior de enfermería del interior del estado de São Paulo, Brasil. Las actividades se organizaron en tres momentos, en tiempo real: *pre-briefing*, *expert-modeling videos* y *debriefing*. Los *expert-modeling videos* sustituyeron al escenario de simulación clínica durante el distanciamiento social impuesto por la pandemia de COVID-19, con el fin de desarrollar la telesimulación. Se utilizaron escalas específicas validadas para analizar la autoconfianza, la autoeficacia y la satisfacción de los estudiantes. El análisis de contenido evaluó las ventajas e inconvenientes del uso de *expert-modeling videos*.

en el estudio participaron 39 estudiantes universitarios. Los participantes mostraron altos niveles de autoconfianza, autoeficacia y satisfacción. Las ventajas de los *expert-modeling videos* se organizaron en las siguientes categorías: Comodidad, Seguridad y continuidad de los estudios y Método de enseñanza eficaz, y las desventajas en Problemas con la tecnología, Distracciones, Falta de contacto personal y Fatiga mental.

las actividades de telesimulación se consolidaron como estrategias que posibilitaron el aprendizaje a distancia en momentos de emergencia de salud por el realismo de la telesimulación, el desarrollo de habilidades en el cuidado de niños y recién nacidos y la articulación entre teoría y práctica.

## Destacados

**(1)** Estrategia innovadora de aprendizaje mediante *expert-modeling videos*.

**(2)** Autoeficacia y autoconfianza de estudiantes universitarios mediante telesimulación en salud infantil.

**(3)** Satisfacción con el uso de la telesimulación en la enseñanza de la enfermería pediátrica y neonatal.

**(4)** Ventajas del uso de *expert-modeling videos* en tiempos de distanciamiento social.

**(5)** La estrategia mostró resultados positivos en el proceso de enseñanza-aprendizaje.

## Introducción

 Con el cierre de las Instituciones de Enseñanza Superior (IES) durante la pandemia de COVID-19 a principios de 2020 ^(1)^, debido a la necesidad de distanciamiento social, los estudiantes y profesores de los cursos de pregrado de enfermería enfrentaron desafíos en el proceso de enseñanza-aprendizaje. En la época, fue necesario buscar nuevas formas de enseñanza para mantener la calidad requerida para el proceso de formación centrado en el cuidado en el individuo y en la comunidad, con vistas a formar un profesional autónomo y proactivo, con pensamiento crítico-reflexivo, razonamiento clínico, capacidad de decisión y discernimiento para actuar en el mercado de trabajo ^(2)^. 

 Como estrategia para continuar con el proceso de enseñanza-aprendizaje, desde el inicio de la pandemia, la mayoría de las actividades se han realizado a distancia, en plataformas en línea como *Google Meet*, *Zoom*, *Skype*, y *Moodle*, entre otras.

 En este contexto, las simulaciones se han destacado como una metodología activa que permite a los estudiantes desarrollar un aprendizaje significativo a través de la vivencia de situaciones realistas en un entorno seguro y libre de riesgos ^(3)^. Los estudiantes pueden participar en el entorno simulado como jugadores de rol u observadores activos; en ambos casos, tienen la oportunidad de aprender habilidades técnicas ^(4)^ y no técnicas, ya sea llevando a cabo acciones o prestando atención a la información disponible en el escenario y a las actividades realizadas por los participantes ^(5)^. Los estudios demuestran que el uso de la simulación tiene un impacto positivo y significativo en la enseñanza de la enfermería cuando se compara con la enseñanza tradicional ^(4, 6 - 9)^. 

 Los estudios que comparan el juicio clínico entre estudiantes participantes, jugadores de rol y observadores han encontrado distintos niveles de análisis y comprensión del caso clínico simulado, pero en general los resultados muestran que la participación como observador también proporciona resultados de aprendizaje significativos ^(5, 10 - 11)^.

 La telesimulación se define como un proceso de enseñanza-aprendizaje que utiliza recursos de telecomunicación y simulación para proporcionar educación, formación y evaluaciones a estudiantes en un lugar externo, cuando existen limitaciones geográficas, por ejemplo ^(12)^. Este modelo de enseñanza incluye *expert-modeling videos*, que se utilizan para preparar a los alumnos poniéndolos en el rol de observadores. Los *expert-modeling videos* pueden ser escenificados por los propios profesores y enfermeros especializados en el tema tratado en el escenario, en un contexto clínico realista en el que los actores realizan tareas o cuidados clínicos de acuerdo con el tema propuesto y los objetivos de aprendizaje ^(13)^. 

 Hay estudios que demuestran que el uso de *expert-modeling videos* en el aula como método de enseñanza-aprendizaje, situando al alumno como observador, mejora su capacidad de juicio clínico ^(5, 14 - 15)^. 

 Los *expert-modeling videos* en telesimulación también se utilizaron para enseñar los cuidados de recién nacidos y niños hospitalizados durante el periodo de cierre de los centros de enseñanza de enfermería, en el que también hubo que suspender las simulaciones clínicas presenciales. 

 El objetivo de este estudio es evaluar la percepción de autoeficacia, autoconfianza y satisfacción de los estudiantes universitarios de enfermería cuando utilizan *expert-modeling videos* como estrategia de aprendizaje para cuidar a recién nacidos y niños hospitalizados durante el distanciamiento social. 

## Método

### Concepción del estudio

 Se trata de un estudio descriptivo cuyo objetivo es conocer la percepción de los estudiantes universitarios de enfermería sobre aspectos de autoconfianza, autoeficacia y satisfacción con el uso de *expert-modeling videos* como estrategia de enseñanza-aprendizaje en telesimulación. 

 Este tipo de estudio pretende observar, describir y documentar aspectos de una situación ^(16)^, en este caso, la utilización de *expert-modeling videos* como estrategia de enseñanza-aprendizaje. 

 Este estudio se realizó de acuerdo con las recomendaciones del *Strengthening the Reporting of Observational studies in Epidemiology* (STROBE). 

### Lugar del estudio

El estudio se realizó en una Institución de Enseñanza Superior (IES) de enfermería de una universidad pública del interior del estado de São Paulo, Brasil. La IES ofrece licenciatura y bachillerato en enfermería, y alrededor de 80 y 50 estudiantes ingresan cada año en los respectivos cursos, totalizando 130 plazas disponibles.

### Descripción del escenario

 Según las buenas prácticas de simulación de la *International Nursing Association for Clinical Simulation and Learning (* INACSL) ^(17)^, las actividades simuladas siguieron las fases de preparación (*pre-briefing* y *briefing*), escenario en acción, en este caso los *expert-modeling videos* y *debriefing* (Figura 1). Con la participación de alumnos y profesores, la sesión informativa previa se celebró en tiempo real, se compartió una pantalla para la transmisión de los *expert-modeling videos* y, a continuación, tuvo lugar la sesión informativa estructurada de forma sincrónica a través de la plataforma *Google Meet*. 

**Figura 1 -:** Etapas para llevar a cabo la telesimulación como estrategia de enseñanza-aprendizaje ^(17)^

 Los *expert-modeling videos* se grabaron previamente en el Centro de Simulación y presentaban cuatro escenarios simulados diferentes, validados y de alta fidelidad, preparados para la práctica con todos los elementos ambientales, materiales y equipos realistas, simuladores de media y alta fidelidad y actores entrenados. Estos cuatro escenarios simulados se desarrollaron y validaron y se han utilizado en cursos durante algunos años, antes de la pandemia; tratan de condiciones y complicaciones clínicas, como la aspiración de la dieta/la deglución, la hipertermia, la deshidratación y la insuficiencia respiratoria, ya que son condiciones/complicaciones clínicas que se dan habitualmente en el contexto de las unidades pediátricas y neonatales. 

### Populación

 Participaron en el estudio estudiantes universitarios de enfermería matriculados en las asignaturas de cuidados a recién nacidos y niños hospitalizados en el curso 2021. Las asignaturas se ofertan semestralmente, en el primer semestre para el grado y la licenciatura en enfermería, con 50 plazas, y en el segundo semestre para el grado en enfermería, con 80 plazas. Durante el curso 2021, los cursos se impartieron a distancia y de forma sincrónica a través de la plataforma *Google Meet*. Se invitó a participar en la investigación a todos los alumnos que realizaron los cursos en ese curso académico. Se excluyeron los ausentes de clase en los días en que la investigadora recogió los datos. 

### Recopilación de datos

 La recogida de datos se llevó a cabo de abril a junio de 2022, por un estudiante de postgrado que acompañó todos los momentos en los que se realizaron las actividades telesimuladas con *expert-modeling videos*. Cada uno de los cuatro escenarios se realizó en diferentes fechas y se organizó en tres momentos: *pre-briefing*, *expert-modeling videos* y *debriefing*, durante el transcurso de los cursos. 

 La elección de no empezar a recoger datos hasta que los sujetos hubieran terminado la investigación se debió a la intención de evitar una relación jerárquica o de autoridad que pudiera limitar la autonomía de los participantes en la investigación ^(18)^. 

Para la recogida de datos, se contactó con los alumnos al inicio de otras asignaturas, con la autorización de los profesores responsables. Al invitarlos, el estudiante de posgrado responsable de la recogida de datos les informó de los objetivos de la investigación, la garantía de participación voluntaria, los riesgos y beneficios de participar en el estudio, haciendo hincapié en que no habría beneficios ni pérdidas en las calificaciones de los alumnos que participaran en la investigación.

 A los que estaban interesados en participar, les entregó un instrumento organizado en dos partes. La primera contenía dos copias del formulario de consentimiento informado, un instrumento de caracterización con datos como el sexo, la edad, el curso (licenciatura o grado y diplomatura), si el participante había tenido contacto previo con la simulación, cómo participaba en la simulación, los tipos de dispositivo tecnológico que utilizaba y un campo abierto para enumerar las ventajas y desventajas de utilizar *expert-modeling videos*. La segunda parte incluyó la Escala de Satisfacción y Autoconfianza del Estudiante en el Aprendizaje, desarrollada por la *National League for Nursing* (NLN), liga dedicada a la excelencia en la enseñanza de la enfermería y validada en Brasil ^(19)^, y la Escala de Autoeficacia ^(20)^, desarrollada por investigadores brasileños a partir de la teoría del aprendizaje social de Bandura. Ambas son autoadministradas y constan de 13 ítems cada una, evaluados mediante una escala tipo Likert con cinco opciones de respuesta (que van de 1 = totalmente en desacuerdo a 5 = totalmente de acuerdo). Cuanto mayor sea la puntuación total en la suma de los ítems, mayores serán los niveles de satisfacción, autoconfianza y autoeficacia percibidos por el estudiante. Participaron 39 estudiantes universitarios, 20 (51,3%) de licenciatura y 19 (48,7%) de diplomatura y licenciatura. 

### Análisis de los datos

 Los datos de caracterización y las puntuaciones de la escala se introdujeron por duplicado en una base de datos preparada con una hoja de cálculo de *Microsoft Excel* ^®^ por dos investigadores de forma independiente. Para el análisis estadístico se utilizó *SPSS Statistics* versión 22 con la ayuda de un estadístico de la institución de enseñanza superior. La estadística descriptiva incluyó el cálculo de la frecuencia, la media, la mediana, la desviación estándar, el mínimo y el máximo de las variables investigadas. 

 Se utilizó el análisis de contenido para analizar las respuestas a las preguntas abiertas sobre ventajas e inconvenientes ^(21)^. Este tipo de análisis permite al investigador cuantificar y calificar los datos. Para ello, se siguieron las etapas de exploración del material, categorización e interpretación. Este proceso fue llevado a cabo por cuatro investigadores del grupo, dos de los cuales se encargaron individualmente de categorizar las ventajas e inconvenientes y de reunirse después para revisarlas, analizar las discrepancias y llegar a un consenso. A continuación, las categorías fueron validadas por otros dos investigadores. Así, las ventajas se organizaron en tres categorías: Conveniencia; Seguridad y continuidad de los estudios; y Método de enseñanza eficaz. Las desventajas se agruparon en cuatro categorías: Problemas con la tecnología; Distracciones; Falta de contacto personal; y Fatiga mental. 

### Consideraciones éticas

 El estudio se realizó de acuerdo con la Declaración de Helsinki y fue aprobado por el Comité de Ética en Investigación de la institución de educación superior (Certificado de Presentación de Apreciación Ética 42613021.2.0000.5393). Todos los participantes involucrados en el estudio fueron informados sobre los objetivos de la investigación y demás aspectos establecidos en las Resoluciones 466 de 2012 ^(22)^ y 510 de 2016 ^(18)^ del CNS. Toda la información fue protegida para garantizar el anonimato y utilizada exclusivamente para fines de la investigación. 

## Resultados

Participaron en el estudio 39 estudiantes, de los cuales el 89,7% (n = 35) eran mujeres, el 7,7% (n = 3) hombres y el 2,6% (n = 1) prefirieron no declarar su sexo. En cuanto a la edad, la media era de 21 años, con un 25,6% (n = 10) de 36 años como máximo y 20 años como mínimo. Todos los estudiantes ya habían tenido contacto previo con el uso de metodologías activas y tecnologías de la información, además de participar en actividades de simulación durante sus estudios de grado antes de la pandemia.

 La Tabla 1 muestra datos sobre la satisfacción de los participantes y su autoconfianza en el aprendizaje. En el eje de satisfacción con el aprendizaje actual, los ítems con mayor frecuencia fueron el 1 (*Los métodos de enseñanza utilizados en esta simulación fueron útiles y eficaces*), con el 76,9% (n = 30) de las respuestas, seguido del ítem 5 (*La forma en que mi profesor enseñó a través de la simulación fue adecuada para mi forma de aprender*), con el 61,5% (n = 24). 

 Los ítems que más destacaron en el eje de autoconfianza fueron el 7 ( *Confío en que esta simulación incluía los contenidos necesarios para dominar el plan de estudios de enfermería pediátrica*) y el 9 ( *Mi profesor utilizó recursos útiles para enseñar la simulación*), ambos con una frecuencia de respuestas del 56,4% (n = 22). 

**Tabla 1 -:** Satisfacción de los estudiantes y autoconfianza en el aprendizaje. Ribeirão Preto, SP, Brasil, 2022

	**Satisfacción con el aprendizaje actual**	**1**	**2**	**3**	**4**	**5**
1	Los métodos de enseñanza utilizados en esta simulación fueron útiles y eficaces.	2,6% (n=1)	7,7% (n=3)	7,7% (n=3)	76,9% (n=30)	5,1% (n=2)
2	El simulacro me proporcionó una gran variedad de materiales didácticos y actividades para promover mi aprendizaje del plan de estudios de enfermería pediátrica.	-	12,8% (n=5)	15,4% (n=6)	51,3% (n=20)	20,5% (n=8)
3	Me gustó la forma en que mi profesor enseñó a través de la simulación.	-	5,1% (n=2)	12,8% (n=5)	59,0% (n=23)	23,1% (n=9)
	**Satisfacción con el aprendizaje actual**	**1**	**2**	**3**	**4**	**5**
4	Los materiales utilizados en la simulación fueron motivadores y me ayudaron a aprender.	-	7,7% (n=3)	23,1% (n=9)	51,3% (n=20)	17,9% (n=7)
5	La forma en que mi profesor enseñó a través de la simulación fue adecuada para mi forma de aprender.	-	7,7% (n=3)	15,4% (n=6)	61,5% (n=24)	15,4% (n=6)
	**Autoconfianza en el aprendizaje**					
6	Tengo confianza en que domino el contenido de la actividad de simulación que me presentó mi profesor.	2,6% (n=1)	17,9% (n-7)	33,3% (n=13)	38,5% (n=15)	7,7% (n=3)
7	Confío en que esta simulación incluía el contenido necesario para dominar el plan de estudios de enfermería pediátrica.	-	12,8% (n=5)	17,9% (n=7)	56,4% (n=22)	12,8% (n=5)
8	Confío en que estoy desarrollando las habilidades y adquiriendo los conocimientos necesarios a partir de esta simulación para realizar los procedimientos necesarios en un entorno clínico.	-	17,9% (n=7)	15,4% (n=6)	48,7% (n=19)	17,9% (n=7)
9	Mi profesor utilizó recursos útiles para enseñar la simulación.	-	2,6% (n=1)	12,8% (n=5)	56,4% (n=22)	28,2% (n=11)
10	Es mi responsabilidad como estudiante aprender lo que necesito saber a través de la actividad de simulación.	-	12,8% (n=5)	28,2% (n=11)	28,2% (n=11)	30,8% (n=12)
11	Sé cómo obtener ayuda cuando no entiendo los conceptos tratados en la simulación.	-	5,1% (n=2)	2,6% (n=1)	46,2% (n=18)	46,2% (n=18)
12	Sé cómo utilizar las actividades de simulación para aprender habilidades.	2,6% (n=1)	5,1% (n=2)	12,8% (n=5)	51,3% (n=20)	28,2% (n=11)
13	Es responsabilidad del profesor decirme lo que necesito aprender sobre el tema desarrollado en la simulación durante la clase.	-	17,9% (n=7)	38,5% (n=15)	30,8% (n=12)	12,8% (n=5)

Al analizar la puntuación total, la media fue de 43,8 y la mediana de 49, con una puntuación máxima de 62, lo que muestra un alto nivel de satisfacción y autoconfianza.

 La Tabla 2 muestra los resultados de la Escala de Autoeficacia. Los ítems que más destacaron en este sentido fueron el 2 (*Confío en mis capacidades*), con una frecuencia de respuesta del 64,1% (n = 25), el 3 (*Cuando decido hacer algo, paso inmediatamente a la acción*), con un 51,3% (n = 20), el 4 (*Afronto bien los problemas inesperados*), también con un 51,3% (n = 20), el 6 (*Encaro dificultades como un desafío*), también con un 51,3% (n = 20), y el 13 (*Me recupero rápidamente después de un fracaso*), con una frecuencia del 51,3% (n = 20). 

**Tabla 2 -:** Escala de autoeficacia (n = 39). Ribeirão Preto, SP, Brasil, 2022

Escala de autoeficacia (EAE)		1	2	3	4	5
1	Soy capaz de realizar con éxito mis proyectos vitales.	-	-	25,6% (n=10)	46,2% (n=18)	28,8% (n=11)
2	Confío en mis capacidades.	-	7,7% (n=3)	17,9% (n=7)	64,1% (n=25)	10,3 (n=4)
3	Cuando decido hacer algo, paso inmediatamente a la acción.	-	5,1% (n=2)	23,1% (n=9)	51,3% (n=20)	20,5% (n=8)
4	Afronto bien los problemas inesperados.	-	15,4% (n=6)	30,8% (n=12)	51,3% (n=20)	2,6% (n=1)
5	Me siento capaz de afrontar bien la mayoría de los problemas que surgen en mi vida.	-	10,3% (n=4)	35,9% (n=14)	38,5% (n=15)	15,4% (n=6)
6	Encaro dificultades como un desafío.	2,6% (n=1)	17,9% (n=7)	12,8% (n=5)	51,3% (n=20)	15,4% (n=6)
7	Me rindo fácilmente en lo que me propongo...	48,7% (n=19)	35,9% (n=14)	5,1% (n=2)	10,3% (n=4)	-
8	Si algo me parece demasiado complicado, ni siquiera lo intento.	59,0% (n=23)	23,1% (n=9)	10,3% (n=4)	7,7% (n=3)	-
9	Me siento inseguro ante el fracaso.	10,3% (n=4)	15,4% (n=6)	25,6% (n=10)	33,3% (n=13)	15,4% (n=6)
10	Me siento abrumado por los fracasos.	20,5% (n=8)	23,1% (n=9)	25,6% (n=10)	25,6% (n=10)	5,1% (n=2)
11	Me siento incapaz de realizar una actividad nueva sin instrucciones.	20,5% (n=8)	25,6% (n=10	28,7% (n=11)	20,5% (n=8)	5,1% (n=2)
12	Puedo decir que he tenido más éxitos que fracasos en mi vida.	-	5,1% (n=2)	30,8% (n=12)	48,7% (n=19)	15,4% (n=6)
13	Me recupero rápidamente después de un fracaso.	-	10,3% (n=4)	35,9% (n=14)	51,3% (n=20)	2,6% (n=1)

En la puntuación total de autoeficacia, hubo una media de 41,5 puntos y una mediana de 42, con una puntuación máxima de 52, lo que muestra un alto nivel en la percepción de los estudiantes.

 Se pidió a los estudiantes que indicaran las ventajas (Figura 2) y los inconvenientes (Figura 3) de utilizar la estrategia de los *expert-modeling videos* para el aprendizaje de la enfermería pediátrica y neonatal. Las respuestas se clasificaron por similitud y se describen a continuación. 

### **Ventajas de los *expert-modeling videos*** **en el proceso de enseñanza-aprendizaje en enfermería pediátrica y neonatal**

 Cuando se les preguntó por las ventajas de utilizar *expert-modeling videos* como estrategia de enseñanza-aprendizaje, los alumnos señalaron aspectos como la comodidad de poder aprender en casa y a salvo de la contaminación por COVID-19, la posibilidad de acercarse a escenarios de práctica incluso a distancia, así como la eficacia de la estrategia para el aprendizaje. Sus declaraciones se clasificaron de la siguiente manera: 

 Los estudiantes señalaron como ventaja la posibilidad de asistir a clase desde la comodidad de su hogar. La categoría Comodidad está presente en afirmaciones como: “*estar en la comodidad del hogar*”, *“ahorra tiempo, porque no tiene que desplazarse a la universidad *”,*”participar desde otra ciudad”*, *“horario de trabajo flexible”*, *“poder revisar el contenido grabado”* e *“posibilidad de verlo de nuevo”*. 

 También se señalaron como ventajas la seguridad de estar protegido frente a la infección por COVID-19 y la posibilidad de mantener el proceso de enseñanza-aprendizaje: *“no perderse ninguna actividad, aunque en período de aislamiento social”*, *“permitió que el curso se desarrollara durante la epidemia”* e *“no contaminar a los colegas”*. 

 La metodología adoptada acercó a los alumnos a los escenarios de la práctica, como se observa en algunas de las afirmaciones: *“más cerca de la realidad”*, “*simulando cómo sería la práctica*”, “*más cerca del escenario de la práctica*” y “*más fácil de entender el contenido teórico y responder a las preguntas*”. 

**Figura 2 -:** Ventajas del uso de *expert-modeling videos* en la enseñanza y el aprendizaje de la enfermería pediátrica y neonatal. Ribeirão Preto, SP, Brasil, 2022

 Además, las declaraciones sugieren que el *expert-modeling videos* puede ser una estrategia eficaz de enseñanza-aprendizaje porque proporciona experiencias diferentes, como: *“preparación para la práctica clínica”*, *“desarrollar sus propias competencias”*, *“utilizar diferentes estrategias con los alumnos”* e *“otra forma de aprender”*. 

 La mayor parte de las afirmaciones positivas estuvieron relacionadas con el método utilizado para continuar el proceso de enseñanza-aprendizaje, señal de que los vídeos de modelización por expertos pueden ser una estrategia poderosa para utilizar no sólo en periodos en los que las clases no pueden ser presenciales, como en el caso de la pandemia, sino también como estrategia adicional a los modelos de aula ya adoptados. En este sentido, han surgido algunas posibilidades de uso de esta herramienta: *“aclaración de dudas”*, *“para procedimientos claros”* e “*proporciona un primer contacto similar a la realidad”*. 

### Inconvenientes de la utilización de *expert-modeling videos* en la enseñanza y el aprendizaje de la enfermería pediátrica y neonatal

Como desventajas, los alumnos señalaron aspectos relacionados con los problemas tecnológicos, la fácil distracción por diferentes estímulos en casa, el cansancio debido a demasiadas clases a distancia y la imposibilidad de practicar los contenidos tratados en clase. Sobre esta base, se presentan a continuación las siguientes categorías:

**Figura 3 -:** Desventajas del uso de *expert-modeling videos* en la formación de enfermería pediátrica y neonatal. Ribeirão Preto, SP, Brasil, 2022

 Se citaron problemas técnicos como desventajas para utilizar el método propuesto, en vista de las afirmaciones sobre la sobrecarga de la red de Internet, tales como “*inestabilidad de Internet”*, *“dificultad para acceder a Internet”, “si se cae la conexión, estorba”,* y en cuanto a la calidad de las imágenes y el audio de los vídeos utilizados: *“depende de un buen Internet para una buena imagen”, “dificultad con la calidad del audio y la imagen”, “el mal micrófono dificultaba la comprensión”* y *“mal encuadre del video”*. 

 A pesar de que la comodidad del hogar se mencionó como una ventaja, los factores de distracción que proporciona este entorno se enumeraron como una desventaja, porque desvían la atención de la actividad docente: “ *fácil dispersión”, “hay más distracciones”, “dispersión y falta de atención por parte de los alumnos”, “distracción como espectador”, “se pierde algo de información”* y *“no retiene tanto la atención”*. Sin embargo, estos inconvenientes pueden compensarse con la posibilidad de revisar el contenido grabado, que también se menciona como una ventaja. 

 Además, los estudiantes señalaron como desventaja la falta de interacción con los contenidos prácticos: “*no sustituye a la práctica/clase presencial”*, *“no desarrollar habilidades como espectador”*, *“no se mancha las manos”* y *“no es práctico para el alumno”*. Este tipo de desventaja es relevante porque puede interferir en la satisfacción y autoconfianza de los estudiantes en el proceso de aprendizaje, lo que no ocurriría si fuera una estrategia para añadir a las diferentes estrategias de enseñanza ya existentes, entre ellas la práctica en escenarios sociales reales. En este sentido, el momento de distanciamiento social provocó en los alumnos una sensación de pérdida del componente clínico de la práctica real. 

 La fatiga mental también se citó como un factor desfavorable a la hora de utilizar la telesimulación con vídeos como estrategia de enseñanza-aprendizaje. Esto se puso de manifiesto en las siguientes afirmaciones: “*fatiga mental ante las clases a distancia”* y”*fatiga debida a muchas actividades a distancia en el ordenador”.* Estas consideraciones se derivan del contexto en el que vivían todos los estudiantes en el momento de la recogida de datos, es decir, el aislamiento social debido a la pandemia de COVID-19, cuando la enseñanza pasó a ser totalmente a distancia. En resumen, se puede destacar que los estudiantes de enfermería consideraron que el método de los *expert-modeling videos* tiene ventajas en situaciones en las que no es posible realizar actividades cara a cara por motivos de seguridad, como en el contexto de una pandemia. Además, se puede utilizar como estrategia para repasar y consultar los contenidos tratados en las asignaturas, pero no sustituye a la enseñanza presencial y a la práctica clínica en entornos sociales. 

## Discusión

 La telesimulación en el proceso de enseñanza-aprendizaje en enfermería neonatal y pediátrica ha demostrado ser atractiva y eficaz, especialmente durante la enseñanza a distancia de urgencias. En los últimos años, la enseñanza sanitaria ha experimentado importantes transformaciones, con énfasis en la educación mediada por la simulación clínica ^(23)^. El uso de la telesimulación para instruir escenarios teóricos y prácticos complejos es un planteamiento relativamente nuevo, como lo es implicar a todos los estudiantes en sus residencias, proporcionándoles una experiencia auténtica e inmersiva, con casos reales y oportunidades de practicar en un entorno clínico ^(24)^. También se ha demostrado que el *debriefing* de la telesimulación es eficaz para aumentar la percepción de la eficacia de la simulación por parte de los estudiantes, por lo que debería integrarse en las experiencias de simulación de enfermería siempre que sea posible ^(25)^.

 El uso de la telesimulación para enseñar un escenario teórico-práctico complejo basado en un caso es relativamente nuevo, al igual que el trabajo con todos los estudiantes situados en sus domicilios, ya que supone proporcionar un modo auténtico e inmersivo, con un caso real y la oportunidad de practicar acciones en un escenario clínico ^(24)^. El propio *debriefing* de telecomunicación ayuda a aumentar la percepción de los estudiantes sobre la eficacia de la simulación y debería incorporarse a las experiencias de simulación de enfermería en la medida de lo posible ^(25)^. 

 En este sentido, en esta investigación, el escenario de telesimulación aportó aspectos de una triangulación de contextos, buscando interactuar con los estudiantes en casa y con los profesores al realizar la telesimulación. Los beneficios de la telesimulación se extienden más allá de los centros de simulación y son válidos cuando existen limitaciones de distancia que impiden una instrucción eficaz y eficiente en una práctica determinada, así como en escenarios complejos que requieren varias repeticiones en el laboratorio ^(26)^. 

 En este estudio, el escenario telesimulado se diseñó para interactuar con los alumnos en casa mientras los profesores dirigían la telesimulación, lo que dio lugar a una triangulación de contextos. Los beneficios de la telesimulación van más allá de los límites de los centros de simulación y son especialmente relevantes en situaciones en las que la distancia dificulta una instrucción eficaz y en escenarios complejos que requieren prácticas repetidas en el laboratorio ^(26)^. La pandemia de COVID-19 ha alterado significativamente la enseñanza y el aprendizaje, y la telesimulación ha surgido como una alternativa crucial para mantener la continuidad en la educación de pregrado, creando experiencias educativas sólidas a distancia que maximizan las oportunidades de aprendizaje ^(27)^. 

 El contexto de la pandemia de COVID-19 ha llevado a cambios en la enseñanza y aprendizaje cotidianos, siendo la telesimulación una alternativa señalada para la continuidad de la formación de pregrado y para crear experiencias educativas a distancia robustas que maximicen las oportunidades de aprendizaje ^(27)^, similar a lo encontrado en este estudio. 

 Otro aspecto es que la mayoría de los estudiantes se mostraron satisfechos con la telesimulación en la modalidad de video modelado por expertos, resultado también identificado en otros estudios, con una percepción de mayor compromiso y estímulo al pensamiento crítico ^(24 - 25)^. También mencionaron la flexibilidad y facilidad para debatir y aprender tanto observando como implicándose activamente. 

 De este modo, se puede atribuir a la telesimulación el mérito de extender los beneficios de las prácticas docentes más allá de los laboratorios y centros de simulación, con diversas ventajas. Algunas de ellas son la accesibilidad, la rentabilidad, el aumento de los conocimientos, el mantenimiento de las actividades, la satisfacción con el aprendizaje, el uso de la simulación en lugares remotos, la mejora de las habilidades, la mejora de la interacción, la interacción interdisciplinar, el aumento de la confianza, la comodidad y el menor riesgo de contaminación ^(6 - 25)^. 

 Los estudios también destacan las limitaciones en la realización de actividades a distancia, debido a fallos de audio ^(24)^ y a distracciones y conexión a Internet limitadas, similares a los retos abordados en este estudio. La creación de escenarios que se asemejan a la realidad, con el fin de captar elementos concretos, desempeñó un papel importante en esta investigación y generó nuevas percepciones como resultado de este proceso de *debriefing*. Este momento de reflexión reveló ideas valiosas para desarrollar perspectivas sobre las prácticas profesionales y la atención sanitaria. El *debriefing* está ampliamente reconocido como eficaz para proporcionar experiencias enriquecedoras y oportunidades de aprendizaje a los estudiantes, y se utiliza en la simulación para la enseñanza de la enfermería con el fin de mejorar las habilidades clínicas y los resultados del aprendizaje ^(28)^. 

 Las experiencias que los estudiantes de enfermería tienen con el uso de tecnologías y estrategias activas durante sus estudios de pregrado son vistas como una posibilidad para diversificar las formas de aprendizaje y, al mismo tiempo, preservar un ambiente seguro y realista. Además, les permiten acercarse a la telesalud, creciente en la práctica clínica y vinculada al aumento de la atención en salud ^(29 - 30)^. Otro factor importante es que las demostraciones de roles a través de vídeos de modelado con expertos también pueden reducir la ansiedad de los estudiantes y mejorar la preparación para las experiencias de aprendizaje simulado ^(29)^. 

 La creación de escenarios realistas desempeñó un papel crucial en la telesimulación, permitiendo una reflexión en profundidad que generó valiosos conocimientos para desarrollar perspectivas sobre las prácticas profesionales y la asistencia sanitaria. El *debriefing* está reconocido como una herramienta eficaz para proporcionar experiencias de aprendizaje enriquecedoras y se utiliza ampliamente en la simulación para la enseñanza de la enfermería con el fin de mejorar las habilidades clínicas y los resultados del aprendizaje ^(28)^. 

 Las experiencias de los estudiantes de enfermería con las tecnologías y las estrategias activas durante sus estudios de grado no sólo diversifican sus formas de aprender, sino que también los preparan para un entorno profesional cada vez más conectado. Además, estas experiencias les acercan a la telesalud, una tendencia creciente en la práctica clínica asociada a los avances en la atención en salud ^(29)^. 

En este estudio se identificaron algunas limitaciones, por ejemplo, se invitó a participar en la investigación a todos los estudiantes matriculados en asignaturas de neonatología y puericultura hospitalizada, de un total de 130, de los cuales sólo el 30% aceptó participar. Esto puede atribuirse al hecho de que, en ese momento, muchos estudiantes ya estaban participando en numerosas actividades a distancia y algunos de ellos estaban sobrecargados con estas demandas de pregrado.

 También es posible que los altos niveles de autoeficacia, autoconfianza y satisfacción medidos en este estudio estén relacionados con la exposición de los participantes a actividades de simulación previas al curso y a la pandemia. La motivación es crucial para un aprendizaje eficaz, y la aplicación de metodologías activas refuerza eficazmente las habilidades adquiridas por los estudiantes durante el proceso de aprendizaje ^(30)^. 

 Cabe destacar que los resultados obtenidos en este estudio muestran la relevancia de discutir y adoptar las tecnologías de la información y comunicación (TIC) en la enseñanza de los cursos de enfermería, ya que son elementos comunes en el día a día de los estudiantes de pregrado hoy en día, además de poder apoyarlos en su autonomía en la búsqueda del conocimiento y la aprehensión de los contenidos ^(31)^. 

## Conclusión

 El estudio mostró que las actividades de telesimulación se consolidaron como estrategias que posibilitaron el aprendizaje en tiempos de emergencia a distancia, debido al realismo de la telesimulación, al desarrollo de habilidades en el cuidado de niños y neonatos y a la articulación entre teoría y práctica. El *pre-briefing* y *debriefing* en tiempo real, ambos por medio de telecomunicaciones (videoconferencia), demostró ser otra estrategia importante para realizar telesimulaciones. 

 En la percepción de los estudiantes, el uso de la estrategia de *expert-modeling videos* en actividades de telesimulación les permitió aprender en tiempos de emergencia a distancia, sentirse cómodos por estar en casa, por el realismo de la telesimulación con la práctica, desarrollar habilidades en el cuidado de niños y recién nacidos y vincular teoría y práctica. Por otro lado, las desventajas fueron los problemas de conexión a la red de Internet, la distracción por estar en casa, el cansancio por el exceso de clases y actividades en línea y la imposibilidad de aplicar los contenidos teóricos en actividades prácticas presenciales en escenarios clínicos reales. 

 A pesar de las desventajas, se destacan los beneficios de la estrategia de *expert-modeling videos* en actividades de telesimulación en cuanto al proceso de enseñanza-aprendizaje de los alumnos, así como el aumento de su nivel de autoconfianza, autoeficacia y satisfacción. Los resultados de este estudio nos permiten identificar las ventajas de utilizar vídeos de modelización de expertos en actividades de telesimulación de forma eficaz y en un entorno virtual seguro, acompañados por profesores facilitadores durante toda la actividad. 

# Translation: Utilização do *expert-modeling videos* em telessimulações no ensino de enfermagem pediátrica e neonatal *

**DOI:** 10.1590/1518-8345.7044.4243

**Published:** 2024-10-23

**Authors:** Mariane Caetano Sulino Gonçalves, Aline Natalia Domingues, Luciana Mara Monti Fonseca, Regina Aparecida Garcia de Lima, Larissa Martiano de Lima, Aline Cristiane Cavicchioli Okido, Juliana Coelho Pina, Adriana Moraes Leite, Lucila Castanheira Nascimento, Maria Cândida de Carvalho Furtado

**Affiliations:** ^1^ Universidade de São Paulo, Escola de Enfermagem de Ribeirão Preto, Centro Colaborador da OPAS/OMS para o Desenvolvimento da Pesquisa em Enfermagem, Ribeirão Preto, SP, Brasil.; ^2^ Bolsista da Coordenação de Aperfeiçoamento de Pessoal de Nível Superior (CAPES), Brasil.; ^3^ Universidade de São Paulo, Escola de Enfermagem de Ribeirão Preto, Departamento de Enfermagem Materno-Infantil e Saúde Pública, Centro Colaborador da OPAS/OMS para o Desenvolvimento da Pesquisa em Enfermagem, Ribeirão Preto, SP, Brasil.; ^4^ Bolsista do Conselho Nacional de Desenvolvimento Científico e Tecnológico (CNPq), Brasil.; ^5^ Universidade Federal de São Carlos, Departamento de Enfermagem, São Carlos, SP, Brasil.; ^6^ Universidade Federal de Santa Catarina, Departamento de Enfermagem, Florianópolis, SC, Brasil.

**Keywords:** Enfermagem Pediátrica, Enfermagem Neonatal, Filme e Vídeo Educativo, Educação em Enfermagem, Treinamento por Simulação, Pesquisa em Educação de Enfermagem

## Abstract

aferir a percepção da autoeficácia, autoconfiança e satisfação de graduandos em enfermagem durante a utilização do *expert-modeling videos* como estratégia de aprendizagem, no período de afastamento social.

estudo descritivo, com graduandos das disciplinas de cuidado ao recém-nascido e à criança hospitalizados de instituição de ensino superior de enfermagem do interior do estado de São Paulo, Brasil. As atividades foram organizadas em três momentos, em tempo real: *prebriefing*, *expert-modeling videos* e *debriefing*. O *expert-modeling videos* substituiu o cenário de simulação clínica durante o afastamento social da pandemia da COVID-19, para desenvolvimento de telessimulação. Para análise da autoconfiança, autoeficácia e satisfação dos estudantes, foram utilizadas escalas validadas específicas. A análise de conteúdo avaliou as vantagens e desvantagens do uso do *expert-modeling videos*.

participaram do estudo 39 graduandos. Foram encontrados níveis altos de autoconfiança, autoeficácia e satisfação entre os participantes. As vantagens do *expert-modeling videos* foram organizadas nas seguintes categorias: Conveniência, Segurança e continuidade dos estudos e Método de ensino eficiente, e as desvantagens em Problemas com tecnologia, Distrações, Ausência de contato pessoal e Fadiga mental.

as atividades de telessimulação consolidaram-se como estratégias que permitiram o aprendizado em tempos de ensino remoto emergencial pelo realismo da telessimulação com desenvolvimento de habilidades no cuidado à criança e ao neonato e articulação entre teoria e prática.

## Destaques

**(1)** Estratégia inovadora de aprendizagem através do uso do *expert-modeling videos*.

**(2)** Autoeficácia e autoconfiança dos graduandos com a telessimulação em saúde da criança.

**(3)** Satisfação com o uso da telessimulação no ensino de enfermagem pediátrica e neonatal.

**(4)** Vantagens da utilização do *expert-modeling videos* em tempos de restrição social.

**(5)** A estratégia demonstrou resultados positivos no processo de ensino aprendizagem.

## Introdução

 Com o fechamento das instituições de ensino superior (IES) durante a pandemia da COVID-19 no início de 2020 ^(1)^, em virtude da necessidade de distanciamento social, estudantes e professores dos cursos de graduação em Enfermagem enfrentaram desafios no processo de ensino-aprendizagem. Na época, foi preciso buscar novas formas de educação para manter a qualidade exigida para o processo de formação voltado para o cuidado centrado no indivíduo e comunidade, com vistas à formação de um profissional autônomo e proativo; com pensamento crítico-reflexivo; raciocínio clínico; capacidade de tomada de decisão; e discernimento para atuar no mercado de trabalho ^(2)^.

 Como estratégia para a continuidade do processo de ensino-aprendizagem, desde o início da pandemia, a maioria das atividades passou a ser realizada remotamente, em plataformas *online* como *Google Meet*, *Zoom*, *Skype*, *Moodle*, dentre outras. 

 Neste contexto, as simulações se destacaram como uma metodologia ativa que possibilita ao estudante desenvolver aprendizagens significativas, oportunizadas pelas vivências de situações realísticas em um ambiente seguro e livre de riscos ^(3)^. A participação do estudante no ambiente simulado pode ser como encenador ou observador ativo; em ambos, ele tem a possibilidade de apreender habilidades técnicas ^(4)^ e não técnicas, ora realizando ações, ora atentando às informações disponíveis no cenário e às atividades realizadas pelos participantes ^(5)^. Estudos demonstram que o uso da simulação apresenta impacto positivo e significativo no ensino de enfermagem, quando comparado à educação tradicional ^(4, 6 - 9)^.

 Já estudos que compararam o julgamento clínico entre estudantes participantes, encenadores e observadores, encontraram níveis variados de análise e compreensão do caso clínico simulado, mas, de modo geral, os resultados mostram que a participação como observador também proporciona resultados significativos de aprendizagem ^(5, 10 - 11)^. 

 A telessimulação é definida como um processo de ensino-aprendizagem que se utiliza dos recursos de telecomunicação e simulação para fornecer educação, treinamentos e avaliações para alunos em local externo, quando há alguma limitação geográfica, por exemplo ^(12)^. Nesse modelo de ensino, encontram-se os *expert-modeling videos*, utilizados como preparo dos alunos, colocando-os no papel de observadores. Os *expert-modeling videos* podem ser encenados pelos próprios professores e enfermeiros especialistas na temática discutida em cena, em um cenário clínico fidedigno, no qual os atores realizam tarefas ou cuidados clínicos, conforme a temática proposta e os objetivos de aprendizagem ^(13)^. 

 Estudos mostram que a utilização de *expert-modeling videos* em sala de aula como método de ensino-aprendizagem, colocando o aluno como observador, melhora suas competências de julgamento clínico ^(5, 14 - 15)^. 

 O *expert-modeling videos* em telessimulação foi também utilizado para ensino dos cuidados com o recém-nascido e a criança hospitalizados, durante o período de fechamento das instituições de ensino de enfermagem, em que as simulações clínicas presenciais também tiveram de ser suspensas. 

 Dessa forma, este estudo tem por objetivo aferir a percepção da autoeficácia, autoconfiança e satisfação de graduandos em enfermagem durante a utilização do *expert-modeling videos* como estratégia de aprendizagem sobre os cuidados com o recém-nascido e a criança hospitalizados, no período de afastamento social. 

## Método

### Delineamento do estudo

 Estudo descritivo, visando aferir a percepção de alunos de graduação em enfermagem quanto aos aspectos de autoconfiança, autoeficácia e satisfação com a utilização do *expert-modeling videos* enquanto estratégia de ensino-aprendizagem em telessimulação. 

 Esse tipo de estudo tem por objetivo observar, descrever e documentar aspectos de uma situação ^(16)^, neste caso, a utilização do *expert-modeling vídeos* como estratégia de ensino-aprendizagem. 

 Este estudo foi reportado segundo as recomendações *Strengthening the Reporting of Observational studies in Epidemiology* (STROBE).

### Local do estudo

O estudo foi realizado em uma Instituição de Ensino Superior (IES) de enfermagem, de uma universidade pública do interior do estado de São Paulo-Brasil. A IES oferece o curso de enfermagem nas modalidades bacharelado e bacharelado e licenciatura, e anualmente ingressam cerca de 80 e 50 alunos nos respectivos cursos, totalizando 130 vagas disponíveis.

### Descrição do cenário

 Segundo as boas práticas em simulação da *International Nursing Association for Clinical Simulation and Learning (* INACSL) ^(^ 17 ^)^ , as atividades simuladas seguiram as etapas de preparação ( *prebriefing* e *briefing*), cenário em ação, neste caso o *expert-modeling videos* e *debriefing* ( Figura 1 ). Com a participação de estudantes e professores, foi realizado o *prebriefing* em tempo real, compartilhada tela para a transmissão do *expert-modeling videos* e, na sequência, ocorreu o *debriefing* estruturado de forma síncrona, por meio da plataforma *Google Meet*. 

**Figura 1 -:** Etapas para realização da telessimulação como estratégia de ensino-aprendizagem ^(^ 17 ^)^

 Os *expert-modeling videos* foram gravados previamente em Centro de Simulação e contaram com quatro diferentes cenários simulados validados, de alta fidelidade e montados para a prática com todos os elementos ambientais realistas, materiais e equipamentos, simuladores de média e alta fidelidade e encenadores treinados. Esses quatro cenários simulados foram desenvolvidos e validados, sendo utilizados nas disciplinas há alguns anos, anteriormente à pandemia; versam sobre condições e intercorrências clínicas, como aspiração de dieta/engasgo, hipertermia, desidratação e insuficiência respiratória, por serem quadros clínicos/intercorrências comumente encontrados nos contextos das unidades pediátricas e neonatais. 

### População

 Participaram do estudo graduandos de enfermagem matriculados nas disciplinas de cuidado com o recém-nascido e a criança hospitalizada, no ano letivo de 2021. As disciplinas são oferecidas semestralmente, no primeiro semestre, para o curso de bacharelado e licenciatura em enfermagem, com 50 vagas, e no segundo semestre para o curso de bacharelado em enfermagem, com 80 vagas. Durante o ano letivo de 2021, as disciplinas foram oferecidas remotamente de forma síncrona, via plataforma *Google Meet*. Todos os alunos que cursaram as disciplinas nesse ano letivo foram convidados a participar da pesquisa. Foram excluídos os ausentes da sala de aula nos dias em que a pesquisadora fez a coleta de dados. 

### Coleta de dados

 A coleta de dados ocorreu de abril a junho de 2022, por uma pós-graduanda que acompanhou todos os momentos em que as atividades telessimuladas com *expert-modeling videos* foram realizadas. Cada um dos quatro cenários foi realizado em datas diferentes e organizado nos três momentos: *prebriefing,* *expert-modeling videos* e *debriefing*, durante o decorrer das disciplinas. 

 A escolha por iniciar a coleta somente após o término das disciplinas foi motivada pela intenção de evitar que uma relação de hierarquia ou de autoridade limitasse a autonomia dos participantes da pesquisa ^(^ 18 ^)^_._

Para a coleta de dados, os alunos foram abordados no início das atividades de outras disciplinas, com a autorização dos professores responsáveis. Ao fazer o convite, a pós-graduanda responsável pela coleta informou sobre os objetivos da pesquisa, garantia de participação voluntária, riscos e benefícios da participação no estudo, reforçando que não haveria benefícios nem prejuízos nas notas dos estudantes que participaram da pesquisa.

 Para os que tiveram interesse em participar, ela entregou um instrumento organizado em duas partes. A primeira continha duas vias do termo de consentimento livre e esclarecido, um instrumento de caracterização com informações como sexo, idade, curso (bacharelado ou bacharelado e licenciatura); se o participante já havia tido contato prévio com simulação, qual a participação na simulação, tipos de dispositivo tecnológico que utilizava e campo aberto para listagem de vantagens e desvantagens na utilização do *expert-modeling videos*. A segunda parte contemplava a Escala de Satisfação de Estudantes e Autoconfiança na Aprendizagem, desenvolvida pela *National League for Nursing* (NLN), liga dedicada à excelência no ensino de enfermagem e validada no Brasil ^(^ 19 ^)^ , e a Escala de Autoeficácia ^(^ 20 ^)^ , desenvolvida por pesquisadores brasileiros com base na teoria de aprendizagem social de Bandura. Ambas são autoaplicáveis e compostas por 13 itens cada, avaliadas a partir de uma escala do tipo Likert contendo cinco opções de resposta (variando de 1= discordo totalmente a 5= concordo totalmente). Quanto maior o escore total na soma dos itens, maiores os níveis de satisfação, autoconfiança e autoeficácia percebidos pelo estudante. Participaram 39 graduandos, sendo 20 (51,3%) do curso de bacharelado e 19 (48,7%) do curso de bacharelado e licenciatura. 

### Análise dos dados

 Os dados de caracterização e os escores das escalas foram digitados duplamente em um banco de dados elaborado via planilha *Excel* da *Microsoft* ^®^ por duas pesquisadoras, de forma independente. Para análise estatística, utilizou-se o *SPSS* *Statistics*, versão 22, com auxílio de um estatístico da instituição de ensino superior. A estatística descritiva incluiu cálculo da frequência, média, mediana, desvio-padrão, mínimo e máximo das variáveis investigadas. 

 Para análise das respostas das questões abertas quanto às vantagens e desvantagens, realizou-se a análise de conteúdo ^(^ 21 ^)^ . Este tipo de análise permite que o pesquisador quantifique e qualifique os dados. Para tanto, seguiram-se as etapas de exploração do material, categorização e interpretação. Esse processo foi realizado por quatro pesquisadoras do grupo, sendo duas individualmente responsáveis por categorizar as vantagens e as desvantagens e, após, se reunirem para revisá-los, analisar as discrepâncias e chegar a um consenso. Na sequência, as categorias foram validadas por outras duas pesquisadoras. Dessa forma, as vantagens foram organizadas em três categorias: Conveniência; Segurança e continuidade dos estudos; e Método de ensino eficiente. Já as desvantagens foram agrupadas em quatro categorias: Problemas com tecnologia; Distrações; Ausência de contato pessoal; e Fadiga mental. 

### Considerações éticas

 O estudo foi conduzido conforme a Declaração de Helsinki e aprovado pelo Comitê de Ética em Pesquisa da instituição de ensino superior com número CAAE (Certificado de Apresentação de Apreciação Ética) 42613021.2.0000.5393. Todos os participantes envolvidos no estudo foram informados sobre os objetivos da pesquisa e demais aspectos previstos nas Resoluções CNS n.º 466 de 2012 ^(^ 22 ^)^ e 510 de 2016 ^(^ 18 ^)^ . Todas as informações foram protegidas para garantia de anonimato e utilizadas exclusivamente para fins de pesquisa. 

## Resultados

Participaram do estudo 39 estudantes, 89,7% (n=35) deles do sexo feminino, 7,7% (n=3) do masculino e 2,6% (n=1) que preferiram não se declarar quanto ao sexo. Com relação à idade, obteve-se média de 21 anos, sendo 25,6% (n=10) com máxima de 36 anos e mínima de 20 anos. Todos os estudantes já haviam tido contato anterior com o uso de metodologias ativas e tecnologias da informação, bem como participado de atividades de simulação durante a graduação antes do período da pandemia.

 A Tabela 1 apresenta dados relativos à satisfação dos participantes e autoconfiança na aprendizagem. No eixo de satisfação com a aprendizagem atual, os itens com maior frequência foram o 1 (*Os métodos de ensino utilizados nesta simulação foram úteis e eficazes*), com 76,9% (n=30) de frequência de respostas, seguido pelo item 5 (*Como meu professor ensinou através da simulação foi adequada para como aprendo)*, com 61,5% (n= 24). 

 Os itens que mais se destacaram no eixo da autoconfiança foram o 7 ( *Estou confiante de que esta simulação incluiu o conteúdo necessário para o domínio do currículo de enfermagem pediátrica*) e o 9 ( *O meu professor utilizou recursos úteis para ensinar a simulação), ambos com frequência de respostas* de 56,4% (n= 22). 

**Tabela 1 -:** Satisfação de estudantes e autoconfiança na aprendizagem. Ribeirão Preto, SP, Brasil, 2022

	**Satisfação com a aprendizagem atual**	**1**	**2**	**3**	**4**	**5**
1	Os métodos de ensino utilizados nesta simulação foram úteis e eficazes.	2,6% (n=1)	7,7% (n=3)	7,7% (n=3)	76,9% (n=30)	5,1% (n=2)
2	A simulação forneceu-me uma variedade de materiais didáticos e atividades para promover a minha aprendizagem do currículo de enfermagem pediátrica.	-	12,8% (n=5)	15,4% (n=6)	51,3% (n=20)	20,5% (n=8)
3	Eu gostei do modo como meu professor ensinou através da simulação.	-	5,1% (n=2)	12,8% (n=5)	59,0% (n=23)	23,1% (n=9)
4	Os materiais utilizados nesta simulação foram motivadores e ajudaram-me a aprender.	-	7,7% (n=3)	23,1% (n=9)	51,3% (n=20)	17,9% (n=7)
5	A forma como meu professor ensinou através da simulação foi adequada para como eu aprendo.	-	7,7% (n=3)	15,4% (n=6)	61,5% (n=24)	15,4% (n=6)
	**A autoconfiança na aprendizagem**					
6	Estou confiante de que domino o conteúdo da atividade de simulação que meu professor me apresentou.	2,6% (n=1)	17,9% (n-7)	33,3% (n=13)	38,5% (n=15)	7,7% (n=3)
7	Estou confiante de que esta simulação incluiu o conteúdo necessário para o domínio do currículo de enfermagem pediátrica.	-	12,8% (n=5)	17,9% (n=7)	56,4% (n=22)	12,8% (n=5)
8	Estou confiante de que estou desenvolvendo habilidades e obtendo os conhecimentos necessários a partir desta simulação para executar os procedimentos necessários em um ambiente clínico.	-	17,9% (n=7)	15,4% (n=6)	48,7% (n=19)	17,9% (n=7)
9	O meu professor utilizou recursos úteis para ensinar a simulação.	-	2,6% (n=1)	12,8% (n=5)	56,4% (n=22)	28,2% (n=11)
10	É minha responsabilidade como aluno aprender o que eu preciso saber através da atividade de simulação.	-	12,8% (n=5)	28,2% (n=11)	28,2% (n=11)	30,8% (n=12)
11	Eu sei como obter ajuda quando não entendo os conceitos abordados na simulação.	-	5,1% (n=2)	2,6% (n=1)	46,2% (n=18)	46,2% (n=18)
12	Eu sei como usar atividades de simulação para aprender habilidades.	2,6% (n=1)	5,1% (n=2)	12,8% (n=5)	51,3% (n=20)	28,2% (n=11)
13	É responsabilidade do professor dizer-me o que eu preciso aprender na temática desenvolvida na simulação durante a aula.	-	17,9% (n=7)	38,5% (n=15)	30,8% (n=12)	12,8% (n=5)

Na análise do escore total, a média chegou a 43,8 e a mediana a 49, numa pontuação máxima de 62, demonstrando alto nível de satisfação e autoconfiança.

 A Tabela 2 , por sua vez, apresenta os resultados da aplicação da Escala de Autoeficácia. Os itens que mais se destacaram neste quesito foram o 2 (*Confio nas minhas habilidades*), cuja frequência de resposta alcançou 64,1% (n= 25), 3 (*Quando decido fazer algo, logo parto para a ação), com 51* ,3% (n=20), 4 (*Lido bem com problemas inesperados*), também com 51,3% (n=20), 6 (*Encaro dificuldades como um desafio*), igualmente com 51,3% (n=20), e 13 (*Recupero-me rapidamente depois de um fracasso*), com frequência de 51,3% (n= 20). 

**Tabela 2 -:** Escala de Autoeficácia (n = 39). Ribeirão Preto, SP, Brasil, 2022

**Escala de Autoeficácia (EAE)**		**1**	**2**	**3**	**4**	**5**
1	Sou capaz de realizar com sucesso meus planos de vida.	-	-	25,6% (n=10)	46,2% (n=18)	28,8% (n=11)
2	Confio nas minhas habilidades.	-	7,7% (n=3)	17,9% (n=7)	64,1% (n=25)	10,3 (n=4)
3	Quando decido fazer algo, logo parto para a ação.	-	5,1% (n=2)	23,1% (n=9)	51,3% (n=20)	20,5% (n=8)
4	Lido bem com problemas inesperados.	-	15,4% (n=6)	30,8% (n=12)	51,3% (n=20)	2,6% (n=1)
5	Sinto-me capaz de lidar bem com a maioria dos problemas que aparecem na minha vida.	-	10,3% (n=4)	35,9% (n=14)	38,5% (n=15)	15,4% (n=6)
6	Encaro dificuldades como um desafio.	2,6% (n=1)	17,9% (n=7)	12,8% (n=5)	51,3% (n=20)	15,4% (n=6)
7	Eu desisto facilmente daquilo que me proponho a fazer.	48,7% (n=19)	35,9% (n=14)	5,1% (n=2)	10,3% (n=4)	-
8	Se algo parece muito complicado, eu nem tento realizá-lo.	59,0% (n=23)	23,1% (n=9)	10,3% (n=4)	7,7% (n=3)	-
9	Sinto-me inseguro diante do fracasso.	10,3% (n=4)	15,4% (n=6)	25,6% (n=10)	33,3% (n=13)	15,4% (n=6)
10	Deixo-me abater diante de fracassos.	20,5% (n=8)	23,1% (n=9)	25,6% (n=10)	25,6% (n=10)	5,1% (n=2)
11	Sinto-me incapaz de realizar uma nova atividade sem a ajuda de instruções.	20,5% (n=8)	25,6% (n=10	28,7% (n=11)	20,5% (n=8)	5,1% (n=2)
12	Posso dizer que na vida tive mais sucessos que fracassos.	-	5,1% (n=2)	30,8% (n=12)	48,7% (n=19)	15,4% (n=6)
13	Recupero-me rapidamente depois de um fracasso.	-	10,3% (n=4)	35,9% (n=14)	51,3% (n=20)	2,6% (n=1)

No escore total para a autoeficácia, houve média de 41,5 pontos e mediana de 42, numa pontuação máxima de 52, demonstrando nível alto, na percepção dos estudantes.

 Foi solicitado aos estudantes que indicassem vantagens (Figura 2) e desvantagens (Figura 3) da utilização da estratégia *expert-modeling videos* na aprendizagem em enfermagem pediátrica e neonatal. As respostas foram categorizadas por similaridade e descritas a seguir. 

### Vantagens do *expert-modeling videos* no processo de ensino-aprendizagem em enfermagem pediátrica e neonatal

 Quando questionados sobre as vantagens da utilização do *expert-modeling videos* como estratégia de ensino-aprendizagem, os estudantes relataram aspectos como: o conforto de poder aprender estando em casa e seguros da não contaminação da COVID-19, a possibilidade de se aproximarem de cenários de prática mesmo que de forma remota, bem como a eficácia da estratégia para a aprendizagem. Suas afirmações foram categorizadas da seguinte forma: 

 Os estudantes identificaram como vantagem a possibilidade de acompanhar as aulas no conforto de suas casas. A categoria Conveniência está presente em afirmações como: “*estar no conforto de casa*”, *“economiza tempo, pois não é preciso se deslocar até a faculdade *”,*”participar de outra cidade”*, *“flexibilidade de horário”*, *“possibilidade de rever o conteúdo gravado”* e *“possibilidade de assistir novamente”.*

 A segurança por estarem protegidos do contágio da COVID-19 e a possibilidade de manterem o processo de ensino-aprendizagem também foram identificadas como vantagens: *“não perder as atividades mesmo em período de distanciamento”*, *“permitiu o andamento do curso durante a epidemia”* e *“não contaminar colegas”.*

**Figura 2:** - Vantagens da utilização do *expert-modeling videos* no ensino-aprendizagem em enfermagem pediátrica e neonatal. Ribeirão Preto, SP, Brasil, 2022

 A metodologia adotada proporcionou aos alunos uma aproximação dos cenários de prática, como observado em algumas falas: “*aproximação com a realidade”*, *“simular como seria a prática”*, *“aproximação do cenário de prática”* e *“facilidade para compreender o conteúdo teórico e sanar dúvidas”*. 

 Além disso, as afirmativas sugerem que o *expert-modeling videos* pode ser uma eficiente estratégia de ensino-aprendizagem por proporcionar diferentes experiências, tais como: *“preparo para prática clínica”*, *“desenvolvimento de habilidades próprias”*, *“utilizar diferentes estratégias com os alunos”* e *“modo diferente de aprender”*. 

 A maioria das afirmações positivas se relacionou ao método utilizado para a continuidade do processo de ensino-aprendizagem, em um sinal de que o *expert-modeling videos* pode ser uma estratégia potente a ser utilizada não somente em períodos nos quais as aulas não puderem ser presenciais, como no caso da pandemia, mas também como uma estratégia adicional aos modelos de aula já adotados. Nesse sentido, emergiram algumas possibilidades para uso dessa ferramenta: *“esclarecimento de dúvidas”*, *“para a clareza dos procedimentos”* e “*proporciona um primeiro contato semelhante à realidade”* . 

### Desvantagens da utilização do *expert-modeling videos* no ensino-aprendizagem em enfermagem pediátrica e neonatal

 Os estudantes apontaram, como desvantagens, aspectos relacionados a problemas tecnológicos, fácil distração por diferentes estímulos em casa, cansaço decorrente do excesso de aulas remotas e impossibilidade de praticar o conteúdo discutido em aula. Com base nisso, as seguintes categorias são apresentadas a seguir (Figura 3): 

**Figura 3:** - Desvantagens da utilização do *expert-modeling videos* no ensino de enfermagem pediátrica e neonatal. Ribeirão Preto, SP, Brasil, 2022

 Problemas técnicos foram citados como desvantagens para utilização do método proposto, tendo em vista as afirmativas sobre a sobrecarga da rede de internet, tais como “*instabilidade da internet”*, *”dificuldade de acesso à internet”*, *”se a conexão cair atrapalha”*, e a respeito da qualidade das imagens e de áudio dos vídeos utilizados:”*depende de uma boa internet para uma boa imagem”*, *“dificuldade com áudio e imagem de qualidade”*, *“microfone ruim dificultava a compreensão”* e *“enquadramento do vídeo ruim”*. 

 Apesar do fato de estar no conforto de casa ter sido referido como uma vantagem, os fatores de distração que este ambiente proporciona foram listados como desvantagem, por tirarem o foco da atividade de ensino: “ *fácil dispersão”*, *“há mais distrações”*, *“dispersão e falta de atenção de alunos”*, *“distração como espectador”*, *“algumas informações se perdem”* e *“não prende tanta atenção”*. Contudo, essas desvantagens podem ser compensadas pela possibilidade de rever o conteúdo gravado, aspecto, inclusive, mencionado como vantagem. 

 Além disso, os estudantes apontaram como desvantagens a falta de interação com o conteúdo prático: “*não substitui a prática/presencial”*, *“não desenvolvimento de habilidades como espectador”*, *“não coloca a mão na massa”* e *“não é prático para o aluno”*. Este tipo de desvantagem é relevante, ao poder interferir na satisfação e autoconfiança no processo de aprendizagem dos estudantes, o que não ocorreria se fosse uma estratégia a somar às diferentes estratégias de ensino já existentes, incluindo prática nos cenários sociais reais. Nesse sentido, o momento do distanciamento social gerou nos estudantes um sentimento de perda do componente clínico da prática real. 

 A fadiga mental também foi citada como um fator de desvantagem na utilização da telessimulação com vídeos como estratégia de ensino-aprendizagem. Tal aspecto foi evidenciado nas seguintes falas: *“cansaço mental diante de aulas remotas”* e *“cansaço devido a muitas atividades remotas no computador”*. Tais considerações decorrem do contexto em que todos os alunos estavam inseridos no período da coleta de dados, ou seja, de isolamento social em virtude da pandemia COVID-19, quando o ensino passou a ser totalmente remoto. Em síntese, pode-se destacar que os estudantes de enfermagem julgaram que o método de *expert-modeling videos* apresenta vantagens em situações nas quais não é possível realizar atividades presencialmente por questões de segurança, como no contexto de uma pandemia. Ademais, pode ser utilizado como estratégia para rever e consultar os conteúdos discutidos nas disciplinas, mas não substitui o ensino presencial e a prática clínica em cenários sociais. 

## Discussão

 A telessimulação no processo de ensino-aprendizagem em enfermagem neonatal e pediátrica mostrou-se atrativa e eficaz, especialmente durante o ensino remoto emergencial. Nos últimos anos, o ensino em saúde passou por transformações significativas, com destaque para a educação mediada por simulação clínica ^(23)^. A utilização da telessimulação para instruir cenários teórico-práticos complexos é uma abordagem relativamente nova, assim como envolver todos os estudantes em suas residências, proporcionando uma experiência autêntica e imersiva, com casos reais e oportunidades de prática em ambiente clínico ^(24)^. O *debriefing* por telessimulação tem se mostrado eficaz para aumentar a percepção dos alunos sobre a eficácia da simulação e deve ser integrado às experiências de simulação de enfermagem sempre que possível ^(25)^. 

 O uso de telessimulação para ensinar um cenário teórico-prático complexo baseado em casos é relativamente novo, bem como trabalhar com todos os estudantes localizados em suas casas, ao envolver proporcionar um modo autêntico e imersivo, com caso real e oportunidade de praticar ações em cenário clínico ^(24)^. O próprio *debriefing* por telecomunicação auxilia no aumento da percepção dos alunos sobre a eficácia da simulação e deve ser incorporado nas experiências de simulação de enfermagem, conforme possível ^(25)^. 

 Nesse sentido, na presente pesquisa, o cenário telessimulado trouxe aspectos de uma triangulação de contextos, buscando interagir com os estudantes em seus domicílios e os docentes na condução da telessimulação. Os benefícios da telessimulação se estendem para além de centros de simulação e são válidos onde há limitações de distância, que impedem a instrução eficaz e eficiente de determinada prática, bem como em cenários complexos que demandam várias repetições em laboratório ^(26)^. 

 Neste estudo, o cenário telessimulado foi projetado para interagir com os estudantes em seus lares, enquanto os docentes conduziam a telessimulação, resultando em uma triangulação de contextos. Os benefícios da telessimulação extrapolam os limites dos centros de simulação e são especialmente relevantes em situações onde a distância dificulta a instrução eficaz e em cenários complexos que requerem prática repetida em laboratório ^(26)^. A pandemia da COVID-19 alterou significativamente o ensino-aprendizagem, e a telessimulação emergiu como uma alternativa crucial para manter a continuidade da formação de graduação, criando experiências educacionais remotas robustas que maximizam as oportunidades de aprendizado ^(27)^. 

 O contexto da pandemia da COVID-19 gerou mudanças no cotidiano do ensino-aprendizagem, sendo a telessimulação uma alternativa que aponta a continuidade da formação de graduação e para criar experiências educacionais remotas robustas, que maximizam oportunidades de aprendizado ^(27)^, semelhante ao encontrado na presente pesquisa. 

 Outro aspecto é que grande parte dos estudantes apontou satisfação com a telessimulação na modalidade do *expert-modeling video*, resultado também identificado em outros estudos, com percepção de maior engajamento e estímulo a pensar criticamente ^(24 - 25)^. Mencionaram-se também a flexibilidade e facilidades na discussão e de aprender tanto observando quanto envolvendo ativamente. 

 Desse modo, pode-se atribuir à telessimulação benefícios do ensino de práticas estendidos para além de laboratórios e centros de simulação, com diversas vantagens. Algumas delas incluem acessibilidade, economia, aumento do conhecimento, manutenção das atividades, satisfação com a aprendizagem, uso da simulação em locais remotos, aprimoramento de habilidades, melhora da interação, interação interdisciplinar, aumento da confiança, conforto e menor risco de contaminação ^(6)^. 

 Estudos também destacam limitações na realização de atividades remotas, pelas falhas do áudio ^(24)^, distrações e conexão de internet limitada, semelhantes aos desafios abordados neste estudo. A criação de cenários que se assemelham à realidade, com o intuito de capturar elementos concretos, desempenhou um papel importante nesta pesquisa e gerou novas percepções como resultado desse processo de *debriefing*. Esse momento de reflexão revelou ideias valiosas para o desenvolvimento de perspectivas sobre práticas profissionais e cuidados de saúde. O *debriefing* é amplamente reconhecido como eficaz para proporcionar experiências enriquecedoras e oportunidades de aprendizado ao estudante, sendo empregado na simulação para o ensino de enfermagem a fim de aprimorar habilidades clínicas e resultados de aprendizagem ^(28)^. 

 As experiências que os estudantes de enfermagem têm com vivências e uso de tecnologias e estratégias ativas durante a graduação são vistas como possibilidade de formas diversificadas de aprender e, ao mesmo tempo, preservar um ambiente seguro e realístico. Além disso, permitem aproximação à telessaúde, que está crescente na prática clínica e ligada ao incremento dos cuidados em saúde ^(29 - 30^. Outro fator importante é que as demonstrações de papéis por meio de vídeos de modelagem com especialistas também podem reduzir a ansiedade dos alunos e melhorar a preparação para experiências de aprendizagem simuladas ^(29)^. 

 A criação de cenários realistas desempenhou um papel crucial na telessimulação, permitindo uma reflexão profunda que gerou *insights* valiosos para o desenvolvimento de perspectivas sobre práticas profissionais e cuidados de saúde. O *debriefing* é reconhecido como uma ferramenta eficaz para proporcionar experiências de aprendizado enriquecedoras e é amplamente utilizado na simulação para o ensino de enfermagem, visando aprimorar habilidades clínicas e resultados de aprendizagem ^(28)^. 

 As experiências dos estudantes de enfermagem com tecnologias e estratégias ativas durante a graduação não só diversificam as formas de aprendizado, mas também os preparam para um ambiente profissional cada vez mais conectado. Além disso, essas experiências os aproximam da telessaúde, uma tendência crescente na prática clínica, associada a avanços nos cuidados de saúde ^(29)^. 

Algumas limitações foram identificadas no presente estudo, por exemplo, foram convidados a participar da pesquisa todos os estudantes matriculados nas disciplinas de cuidado com o recém-nascido e criança hospitalizados, em um total de 130, dos quais somente 30% aceitaram participar. Isso pode ser atribuído ao fato de que, na época, muitos estudantes já participavam de inúmeras atividades remotas e alguns deles estavam sobrecarregados com essas demandas da graduação.

 É possível também que os níveis altos de autoeficácia, autoconfiança e satisfação aferidos no presente estudo estejam relacionados à exposição dos participantes nas atividades de simulação anteriores à disciplina e à pandemia. A motivação é crucial para uma aprendizagem eficaz, e a aplicação de metodologias ativas fortalece de maneira efetiva as habilidades adquiridas pelos estudantes durante o processo de aprendizagem ^(30)^. 

 Vale destacar que os resultados obtidos neste estudo evidenciam a relevância de discutir e adotar tecnologias de informação e comunicação (TIC) no ensino dos cursos de enfermagem. Trata-se de elementos comuns no cotidiano dos alunos de graduação na atualidade, bem como podem apoiá-los na autonomia na busca de conhecimento e a apreensão de conteúdo ^(31)^. 

## Conclusão

 O estudo permitiu evidenciar que as atividades de telessimulação se consolidaram como estratégias que permitiram o aprendizado em tempos de ensino remoto emergencial pelo realismo da telessimulação com desenvolvimento de habilidades no cuidado à criança e ao neonato e articulação entre teoria e prática. A realização do *prebriefing* e *debriefing* em tempo real, ambos por meio das telecomunicações (videoconferência), revelou-se outra importante estratégia na execução de telessimulações. 

 Na percepção dos estudantes, o uso da estratégia *expert-modeling videos* em atividades de telessimulação permitiu: o aprendizado em tempos de ensino remoto emergencial, conforto por estarem em seus domicílios, pelo realismo da telessimulação com a prática, desenvolvimento de habilidades no cuidado à criança e ao neonato e articulação entre teoria e prática. Já como desvantagens, foram citados problemas de conexão à rede de internet, distração por estarem em domicílio, cansaço decorrente do excesso de aulas e atividades remotas e a impossibilidade de aplicar o conteúdo teórico a atividades práticas presenciais nos cenários clínicos reais. 

 Apesar das desvantagens, os benefícios da estratégia *expert-modeling videos* em atividades de telessimulação se sobressaem no que tange ao processo de ensino-aprendizagem dos estudantes, assim como ao aumento do nível de autoconfiança, autoeficácia e satisfação. Os resultados deste estudo permitem identificar vantagens no uso do *expert-modeling video* em atividades de telessimulação efetivamente e em um ambiente virtual seguro, com acompanhamento de professores facilitadores durante toda a atividade.
